# Expression of 6-Cys Gene Superfamily Defines *Babesia bovis* Sexual Stage Development within *Rhipicephalus microplus*

**DOI:** 10.1371/journal.pone.0163791

**Published:** 2016-09-26

**Authors:** Heba F. Alzan, Audrey O. T. Lau, Donald P. Knowles, David R. Herndon, Massaro W. Ueti, Glen A. Scoles, Lowell S. Kappmeyer, Carlos E. Suarez

**Affiliations:** 1 Department of Veterinary Microbiology and Pathology, College of Veterinary Medicine, Washington State University, Pullman, WA, United States of America; 2 Parasitology and Animal Diseases Department, National Research Center, Dokki, Giza, Egypt; 3 Animal Disease Research Unit, United States Department of Agricultural—Agricultural Research Service, Pullman, WA, United States of America; 4 The National Institute of Allergy and Infectious Diseases, 5601 Fishers Lane, MSC 9823, Bethesda, MD, United States of America; University of Minnesota, UNITED STATES

## Abstract

*Babesia bovis*, an intra-erythrocytic tick-borne apicomplexan protozoan, is one of the causative agents of bovine babesiosis. Its life cycle includes sexual reproduction within cattle fever ticks, *Rhipicephalus* spp. Six *B*. *bovis* 6-Cys gene superfamily members were previously identified (*A*, *B*, *C*, *D*, *E*, *F*) where their orthologues in *Plasmodium* parasite have been shown to encode for proteins required for the development of sexual stages. The current study identified four additional 6-Cys genes (*G*, *H*, *I*, *J*) in the *B*. *bovis* genome. These four genes are described in the context of the complete ten 6-Cys gene superfamily. The proteins expressed by this gene family are predicted to be secreted or surface membrane directed. Genetic analysis comparing the 6-Cys superfamily among five distinct *B*. *bovis* strains shows limited sequence variation. Additionally, *A*, *B*, *E*, *H*, *I* and *J* genes were transcribed in *B*. *bovis* infected tick midgut while genes *A*, *B* and *E* were also transcribed in the subsequent *B*. *bovis* kinete stage. Transcription of gene *C* was found exclusively in the kinete. In contrast, transcription of genes *D*, *F* and *G* in either *B*. *bovis* infected midguts or kinetes was not detected. None of the 6-Cys transcripts were detected in *B*. *bovis* blood stages. Subsequent protein analysis of 6-Cys A and B is concordant with their transcript profile. The collective data indicate as in *Plasmodium* parasite, certain *B*. *bovis* 6-Cys family members are uniquely expressed during sexual stages and therefore, they are likely required for parasite reproduction. Within *B*. *bovis* specifically, proteins encoded by 6-Cys genes *A* and *B* are markers for sexual stages and candidate antigens for developing novel vaccines able to interfere with the development of *B*. *bovis* within the tick vector.

## Introduction

Bovine babesiosis is caused by an intra-erythrocytic infection [1] with the protozoan parasite *Babesia bovis*, which is transmitted primarily by the cattle fever tick, *Rhipicephalus (Boophilus*) *microplus*. Bovine babesiosis is a significant health and economic issue for the cattle industry, due to the fact that infections cause high mortality and morbidity rates worldwide [2]. Current control strategies include live blood-based vaccines using attenuated strains of *B*. *bovis*, babesicidal drugs (e.g. imidocarbdipropionate), other means such as containment of tick vector populations through acaricide applications, and/or the use of tick-based vaccines for the control of cattle fever ticks, animal infection and pasture management are also employed [3].

Each current control strategy has pitfalls. Although live attenuated *Babesia* vaccines are effective at preventing the disease, their limited use is due to their potential for virulence reversion, the costly cold chains requirement for vaccine preservation during transportation and storage, and the risks of contamination by other blood-borne pathogens [1, 3].

In addition, live attenuated vaccines do not prevent infection by or transmission of wild-type strains. Babesial chemotherapeutics and tick control via acaricide applications are also limited due to their high cost, development of parasite and tick resistance as well as the addition of toxic residues to the food chain [4–6]. Furthermore, acaricide use in regions in which enzootic stability is maintained may lead to higher risk of disease with declining population immunity from reduced tick-borne parasite transmission [7]. Thus, the development of novel methodologies is required to provide population immunity. Novel vaccine approaches necessitate the identification of antigens crucial for the completion of parasite’s life cycle and/or transmission.

*B*. *bovis* has a complex life cycle that involves cattle and its definitive host, *Rhipicephalus* ticks. Asexual stages replicate in the mammalian erythrocytes leading to life threatening anemia during acute stage of infection. When a tick acquires a blood meal from an infected bovine host, infected erythrocytes are ingested and parasites develop into male and female gametes (sexual stages) in the tick midgut [8, 9]. The gametes fuse and form diploid zygotes invading the midgut epithelial cells to become motile kinetes. The kinetes invade ovary tissues and ultimately develop into infectious sporozoites in the salivary glands of the larval offspring. Although there is considerable knowledge concerning molecular and morphological aspects of *B*. *bovis* erythrocyte stages [10], very little is known about sexual stage development within tick midgut epithelial cells or the lumen. The major obstacle stems from technical difficulties in obtaining sufficient amount for the manipulation of the tick midgut tissues and the lack of an *in vitro* system for induction of sexual stage development. In contrast, considerably more is known about *B*. *bigemina*, where gametocytes were first characterized by Koch, 1906 [11], and a method for *in vitro* induction of sexual stages was established [12]. Consequently, CCp protein family members are just one of few *B*. *bigemina* sexual stage molecular markers that have so far been identified [13]. Despite the close phylogenetic relationship between these two *Babesia* species, orthologous *B*. *bovis* CCp proteins are not detected in the tick stages.

A genomic search of phylogenetically related *Plasmodium falciparum*, the causative agent of human malaria, revealed a six-cysteine (6-Cys) gene family [14–19]. The proteins expressed from *P*. *falciparum* 6-Cys genes are known to function in the recognition and adhesion of male and female gametes [20, 21]. Three of the *Plasmodium* 6-Cys genes (Pfs230, Pf48/45 and Pfs47) are expressed in gametes. Proteins Pfs230 and Pf48/45 are proposed to be *Plasmodium* transmission blocking vaccine candidates [22] as both proteins not only play an essential role in parasite fertilization, they are also accessible to antibody generated by the vaccination of a vertebrate host with these specific antigens. Protein Pfs47, on the other hand, dampens the mosquito’s immune system and promotes parasite survival [23]. *In silico* comparative genomic analysis reveals the presence of 6-Cys domain-containing protein-coding orthologous genes in *B*. *bovis* (Bbo 6-Cys) [24]. Based on their sequence similarity and domain conservation, we hypothesize that these orthologous genes in *B*. *bovis* are involved in parasite sexual development.

A promising approach is the development of transmission blocking vaccines (TBV) by targeting parasite’s antigens that are essential for completion of the parasite life cycle. Immunologic targeting of parasite antigens that are involved in transmission has been demonstrated to be an effective strategy for blocking transmission of *Plasmodium* [25]. Until recently, the development of TBVs has been hindered in part by the failure to identify and characterize *B*. *bovis* tick-stage specific antigens. Therefore, the specific goal of this study was to identify potential *B*. *bovis* antigens for the design of a transmission blocking vaccine. This work reports the characterization of 6-Cys gene expression within the *B*. *bovis* life cycle where the number, conservation and expression pattern of 6-Cys genes were determined. Interestingly, unexpected differential expression patterns of 6-Cys genes were discovered at different *B*. *bovis* life cycle stages.

## Material and Methods

### Parasite strain and *in vitro* cultivation

The *B*. *bovis* parasites were grown in long term microaerophilous stationary-phase culture as previously described [26, 27] The T3Bo strain (*B*. *bovis* Texas S74-T3Boderived from S1-T2Bo, [28]) and the Mo7 biological clonal strain of *B*. *bovis* [27, 29] were maintained as cryopreserved stabilates in liquid nitrogen [30]. Genomic DNA from *B*. *bovis* Australian (T strain) and Argentina (L17) virulent and attenuated strain pairs were kindly provided by Dr. Audrey Lau [31]. *B*. *bovis* T3Bo strain-infected bovine blood was used as controls in the reverse transcription-polymerase chain reaction (RT-PCR).

### *In silico* genes identification by genomic search and bioinformatics analysis

New members of the Bbo 6-Cys gene family were identified using TBLASTN search against the available Conserved Domain Database of NCBI (http://www.ncbi.nlm.nih.gov/cdd) and the published *B*. *bovis* genome (www.vetmed.wsu.edu/research_vmp/Babesia-bovis/) [32]. Multiple amino acid sequence alignments and calculation of sequence identities among the Bbo 6-Cys family members and members of the *Plasmodium* 6-Cys protein family which include Pfs230 (AAG12332), Pf48/45 (XP_001350181) and Pfs47 (XP_001350182) [33] were carried out using Clustal Omega Multiple Alignment (http://www.ebi.ac.uk/Tools/msa/clustalo/). Phylogenetic tree prediction generated by Phylogeny.fr. This tree prediction is based in an approximate likelihood-ratio test (aLRT) as an alternative to nonparametric bootstrap and Bayesian estimation of branch support [34, 35]. Sequence identity and similarity calculations were conducted via http://www.genome.jp and http://www.bioinformatics.org, respectively. Domain prediction of Bbo 6-Cys family protein sequences was performed using the SMART program (http://smart.embl-heidelberg.de and http://pfam.xfam.org/search. Trans-membrane domains and signal peptides were predicted using the Transmembrane Hidden Markov Model package 2 (TMHMM2) (http://www.cbs.dtu.dk/services/TMHMM-2.0). The detection of glycosylphosphatidylinositol (GPI) anchor was predicted using an online GPI prediction server (http://mendel.imp.ac.at/gpi/gpi_server.html. Motifs prediction was performed using http://meme-suite.org/. Proteins translocation and subcellular localization predictor, Cello v2.5 (http://cello.life.nctu.edu.tw/) was also used.

### Genomic DNA extraction and amplification of Bbo 6-Cys genes from different geographically distinct strains

DNA was extracted from whole blood collected from the T3Bo, Mo7 clonal line, Argentina (L17) and Australian (T) strains[31]. Primers used to amplify the whole open reading frames of all members of the Bbo 6-Cys family were designed manually (S1 Table). Amplification of r*ap-1* gene was used as a PCR control. *Rap*-1 gene forward and reverse primers are BoFN: 5'- TCA ACA AGG TAC TCT ATA TGG CTA CC -3' and BoRN: 5'- CTA CCG AGC AGA ACC TTC TTC ACC AT -3'. Quantitation of gDNA was determined using a Nano-drop spectrophotometer.

### Cloning and sequencing of Bbo 6-Cys genes

Primers used for sequencing all the Bbo 6-Cys are provided in S1 Table. All the PCR products derived from each gene and from each *B*. *bovis* strain were cloned in Topo 2.1 vector, transformed into competent *Escherichia coli* TOP10 cells and cultured in antibiotic selection media based on manufacture’s guidelines (Invitrogen). Three colonies per amplification for each strain were randomly selected. The purified plasmids were sequenced using M13 forward and reverse primers as well as the gene specific primers designed internal of the genes (S2 Table) for the sequencing procedure. New 6-Cys family gene sequences in different *B*. *bovis* strains were deposited in the Genbank with the accession numbers (S3 Table).

### Polymorphism and phylogenetic analysis

The complete gDNA sequences for 6-Cys family member were compared among five geographically distinct strains. Strain-specific single nucleotide polymorphisms (SNPs) were then estimated in order to calculate the ratio of synonymous to non-synonymous changes. To estimate ω (dN/dS ratio), “SNAP” was used (http://hcv.lanl.gov/content/sequence/SNAP/SNAP.html). The parameters were set up as follows: ω >1 indicates positive selection, as the selection has caused some amino acid substitution; ω<1 indicates occurrence of purifying selection and a high degree of sequence conservation [36]. Clustal omega was used for DNA sequences alignment and the program Phylogeny.fr was used to generate phylogenetic relationship among 6-Cys family member. Nucleotide substitutions were manually calculated.

### Experimental *B*. *bovis* infection in bovine and ticks and cDNA synthesis

A spleenectomized Holstein calf approximately 4 months of age and determined to be babesiosis free by competitive enzyme-linked immunosorbent assay and PCR targeting RAP-1[37] was used to synchronize female tick acquisition feeding with ascending *B*. *bovis* parasitemia. Approximately 40,000 *R*. *microplus* larvae (La Minita strain) were placed in a cloth patch on splenectomized calf. At 14 day post-tick application when 1% of the ticks had molted to adults, the calf was inoculated intravenously with *B*. *bovis* T3Bo strain stabilate contained 10^7^ infected erythrocytes. The peak *B*. *bovis* parasitemia coincided with the peak drop of replete female ticks. The replete female ticks were collected and incubated at 26°C in 96% relative humidity to allow *B*. *bovis* sexual stage development. Representative samples of the infected replete female ticks were collected each day post incubation and dissected individually in sterile 1x phosphate buffered saline (PBS). Tick midguts were collected from each individual tick and stored in Trizol at -80°C (Invitrogen).

Blood from the infected animal was collected at peak parasitemia (~ day 12) for DNA and RNA extractions using Trizol method. For quality verification, the Nanodrop was used to measure the OD 360/280. Total RNA was isolated from (i) cultured blood stage parasites from *B*. *bovis* T3Bo, culture Mo7 clonal line, Argentina L17 (virulent), and two Australian (T) strains (virulent and attenuated) [31], (ii) blood of the calf experimentally infected with *B*. *bovis*, (iii) *B*. *bovis* infected tick midguts that were dissected from ticks incubated after repletion for 24, 48, and 72 h. RNA concentrations were determined using a Nano-drop spectrophotometer. Extracted RNA was treated with RNase inhibitor (Roche) and RNase-free DNase (Turbo DNA-free from Ambion) for 30 min at 37°C. The cDNA was synthesized using the SuperScript II RNase H reverse transcriptase (RT) first strand synthesis system (Invitrogen), according to the manufacturer's protocol using random hexamer primers. Gene-specific primers were used to amplify each gene of the 6-Cys family member (S1 Table) from different developmental stages of the *B*. *bovis*. All amplicons were confirmed by sequencing (data not shown).

### Synthetic peptide design and subsequent polyclonal antibody generation

Synthetic peptides ranging from 15 to 24 amino acids (aa) representing protein-specific sequences of the Bbo 6-Cys proteins A and B were synthesized (S4 Table) to produce polyclonal antibodies. The peptides were conjugated to keyhole limpet hemocyanin (KLH) and used in the immunization of rabbits (BioSynthesis). The resulting immune sera were titrated by ELISA and used in subsequent immunoblot assays. Peptides that range between aa 583–598 and aa 287–306 were included in the western blot analysis as controls for 6-Cys proteins A and B, respectively.

### Detection of Bbo 6-Cys proteins by immunoblot assays

Protein lysates were prepared from *B*. *bovis*-infected erythrocyte culture, infected tick midguts and hemolymph with *B*. *bovis*. Infected midgut and hemolymph samples were collected in the same manner as previously described in the experimentally *B*. *bovis* infection. Female ticks were collected from a calf infected with T3Bo and incubated as described above. Infected female ticks were dissected at days 2, 3 and 4 post-incubation and midguts were collected and stored in 1x PBS with proteinase inhibitors cocktail (Roche) at -80°C. Infected hemolymph was collected from infected replete female ticks at day 7 post-repletion, in, into Hank’s balanced salt solution (Sigma) and kept at -80°C. Samples were washed and centrifuged three times followed by the addition of cell lysis buffer with proteinase inhibitors cocktail to be used in the immunoblot assay. Peptides for 6-Cys proteins A (aa 583–598) and B (aa 287–306) were included as controls in the immunoblot. Briefly, upon the addition of 5x SDS-PAGE sample buffer (GenScript), samples were boiled for 5 min and then sonicated for 2 min with 20 sec interval. Approximately 10 μl of total lysate were loaded and separated in 4–20% Mini-PROTEAN® TGX™ Precast Gels (Bio-Rad). The separated proteins were transferred to nitrocellulose membranes and blocked in 0.01% 1x PBS Tween 20 containing 5% non-fat milk for 1 h at room temperature (RT). The membranes were incubated with shaking for 1h at RT with an appropriate primary antibody against Bbo 6-Cys A and B (1:100 dilutions). Monoclonal RAP-1 antibodies were used to detect RAP-1 protein during *in vitro* cultured *B*. *bovis* blood stage [30] as well as pre-immune rabbit serum as positive and negative controls, respectively. The membranes were washed three times in 0.01% 1x PBS Tween 20 for 1h. After washing, membranes were incubated with an appropriate HRP conjugated secondary antibody (goat anti-mouse and goat anti-rabbit IgG (H+L)[KPL] with 1:5000 dilution) for 1h at RT. Antibody reactivity was developed using chemiluminescent HRP antibody detection reagents(KPL, Gaithersburg, Maryland, USA).

## Ethical Statement

All calves used in this study were purchased from the University of Idaho Dairy. Animals were housed in a climate controlled barn with natural light cycle (windows) fed twice per day (morning and afternoon) and provided water ad lib. During tick feedings cattle were housed in stanchions. The calves were monitored during the experimental procedure at least twice daily, but were under constant observation during working hours. The clinical signs of the infection with *Babesia bovis* include increased fever, marked decrease in the hematocrit, and neurological signs. The pre-patent period is about seven to eight days and signs of acute infection typically appear about 9–10 days post-infection. We monitored all clinical parameters during the course of the infection. The experiments were humanely terminated if the animals experience increased fever of >104°F for more than 3 days or the hematocrit goes below 15%. It was anticipated that the experiments would be terminated before the animals experience acute signs of the disease. However, clinical signs did not warrant palliative treatment during the course of the experiment, Banamine (analgesic) was available for treatment if it had been deemed to be necessary. Towards the end of the experiment animals were humanely euthanized by intra venous administration of Sodium pentobarbital at a dose of 10 ml per 100 lbs body weight (as per label instructions). In most cases, cattle are first sedated with Xylazine and brought to a recumbent position prior to euthanasia. Euthanasia is always performed by a fully trained technician. This study was approved by the Institutional Animal Care and Use Committee of the University of Idaho, Moscow, Idaho (protocol #2016–20), in accordance with institutional guidelines based on the U.S. National Institutes of Health (NIH) Guide for the Care and Use of Laboratory Animals.

## Results

### *In silico* characterization of novel Bbo 6-Cys genes in the context of complete family

**Fig 1:**
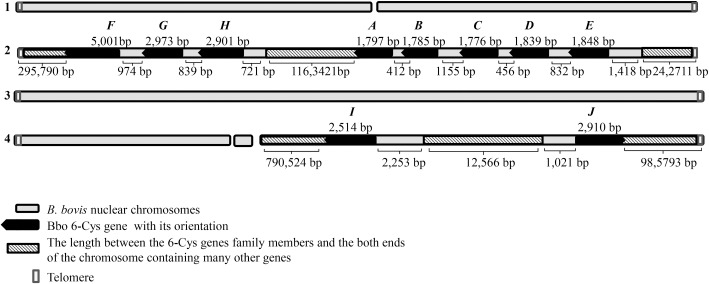
Bioinformatic analysis of Bbo 6-Cys family genes. Schematic representation of the locations and relative orientations of the Bbo 6-Cys genes family members into the four *B*. *bovis* nuclear chromosomes. The lengths of the ORFs, the intergenic regions among them, and their distance from the chromosomes ends are indicated in bp. The size of the genes is not represented in a proportional scale.

Six 6-Cys gene-family members were previously identified in *B*. *bovis* and are designated as Bbo 6-Cys *A-F* [24]. Four additional Bbo 6-Cys genes (GenBank accession numbers: XP_ II001190, XP_II001120, XP_IV007390 and XP_IV007480) were identified by TBLASTN searches using *Plasmodium* 6-Cys domain. They are designated as Bbo 6-Cys *G*, *H*, *I* and *J*. The exact chromosome localizations, lengths, and features of the ten *B*. *bovis* 6-Cys genes are detailed in Fig 1 and Table 1. Briefly, Bbo 6-Cys genes *A-H* and *I-J* are on chromosomes 2 and 4, respectively. The previously characterized Bbo 6-Cys *A-E* genes cluster together and are tandemly arranged in a head to tail organization [24]. The newly identified Bbo 6-Cys *F-H* genes form a separate cluster, and are oriented in the same manner as A-E on the opposite end of chromosome 2. The two clusters, A-E and F-H are separated by 1,163,421 bps (Fig 1). In contrast, Bbo 6-Cys *I* and *J* genes are located on chromosome 4, separated by 12,566 bps and are oriented head to head (Fig 1).

**Table 1:** Predicted features of the *B*. *bovis* 6-Cys family genes and their putative proteins.

	Locus tag	Gene range in the *B*. *bovis* genome	DNA/Protein[bp/aa]	Exon count	Annotation	No. of 6-Cys domain	Predicted MW[KDa]/PI
A	BBOV_II006600	1473394..1475190	1797/598	One	Hypothetical protein	2	66.73/7.46
B	BBOV_II006610	1475602..1477386	1785/594	One	Hypothetical protein	2	67.88/7.7
C	BBOV_II006620	1478541..1480316	1776/591	One	Hypothetical protein	2	87.86/7.85
D	BBOV_II006630	1480772..1482610	1839/612	One	Hypothetical protein	2	69.51/6.79
E	BBOV_II006640	1483442..1485289	1848/615	One	Putative protein membrane protein	2	70.28/8.82
F	BBOV_II001180	295791..301497	5707/1689	Nine	Hypothetical protein	3	187.27/6.90
G	BBOV_II001190	302471..305443	2973 /990	One	Hypothetical protein	2	113.05/6.46
H	BBOV_II001200	306282..309182	2901 /966	One	Hypothetical protein	2	108.69/6.76
I	BBOV_IV007390	790524..793037	2514 /837	One	Hypothetical protein	1	96.16/6.16
J	BBOV_IV007480	808875..811784	2910 /969	One	Putative protein membrane protein	1	108.96/8.10

The sizes of Bbo 6-Cys family gene members vary with the smallest being Bbo 6-Cys gene *A* and the largest, Bbo 6-Cys gene *F*. The gene sizes of the *A-E* cluster range from 1,797 bp to 1,848 bp while sizes of the *H-F* cluster are between 2,901 bp to 5,707bp. *I* and *J* are 2,514 bp and 2,910 bp respectively (Fig 1). The less typical gene of the *B*. *bovis* 6-Cys gene family is gene *F*, which was originally annotated as a 5,707 bp gene encoding for a putative 1,689 amino acid protein, uniquely contains eight introns (S1 Fig and Table 1).

Using the same *in silico* analysis on related apicomplexan genomes, we also investigated potential 6-Cys orthologues and determined the presence of the sexual stage antigen s48/45 domain in 82 protein-coding genes distributed in the five chromosomes of *B*. *bigemina* [Bond strain], two in *B*. *microti* [R1], one in *Theileria equi* [WA strain] and two in both *T*. *parva* [Muguga strain] and *T*. *annulata* [Ankara strain] (S5 Table).

### Polymorphism analysis of Bbo 6-Cys family members among different geographically strains

To estimate the degree of polymorphisms of the 6-Cys gene family members, we compared the DNA sequences of all ten Bbo 6-Cys genes among five distinct *B*. *bovis* strains [Tx-attenuated and Tx-virulent, Argentinian L17, Australian T-attenuated and T-virulent]. Sequence comparisons showed that all 6-Cys genes are highly conserved among the five *B*. *bovis* strains (96 to 100% aa identity) [GenBank accession numbers for each sequence is shown in S3 Table]. Sequence comparisons of 6-Cys genes were also used to find out if immune selection pressure in the bovine host affects this gene family. We calculated the non-synonymous (dN) to synonymous (dS) polymorphism among sequenced 6-Cys genes from these *B*. *bovis* strains (Table 2) with the parameter, ω, (ω = dN/dS), as an indicator of potential selection pressures. In all cases, a ω of less than 1 was obtained suggesting that it is improbable that these gene family members are under selective forces such as those from the host immune system. These data are consistent with a low or negligible rate of exposure of the *B*. *bovis* 6-Cys proteins to the host immune selection. These data also indicate the conservation of *B*. *bovis* 6-Cys family members throughout different *B*. *bovis* strains implies an essential function.

**Table 2:** Demonstration of the polymorphism and the average SNPs among the *B*. *bovis* 6-Cys gene family members among different distinct strains.

	**A**	**B**	**C**	**D**	**E**	**F**	**G**	**H**	**I**	**J**
**NS**	45	48	54	45	18	170	73	83	101	122
**Non-synonymous substitutions**	26	20	19	20	6	71	31	36	59	57
**Synonymous substitutions**	19	28	35	25	12	99	42	47	42	65
**Average dN**	0.0140	0.0079	0.0156	0.0091	0.0028	0.0091	0.0078	0.0091	0.0176	0.0164
**Average dS**	0.0376	0.0488	0.0290	0.0352	0.0168	0.0487	0.0387	0.0468	0.0422	0.0598
**Average dN/dS ratio**	0.372	0.16	0.53	0.25	0.16	0.18	0.2	0.19	0.4	0.27
**pN/pS**	0.3	0.0167	0.53	0.26	0.16	0.19	0.2	0.19	0.4	0.28

NS: nucleotide substitution; dN: non-neutral; pN: non-neutral polymorphism divergence; dS: neutral divergence; pS: neutral polymorphism.

### *In silico* protein analysis and phylogenetic relationships among the Bbo 6-Cys family members

**Fig 2:**
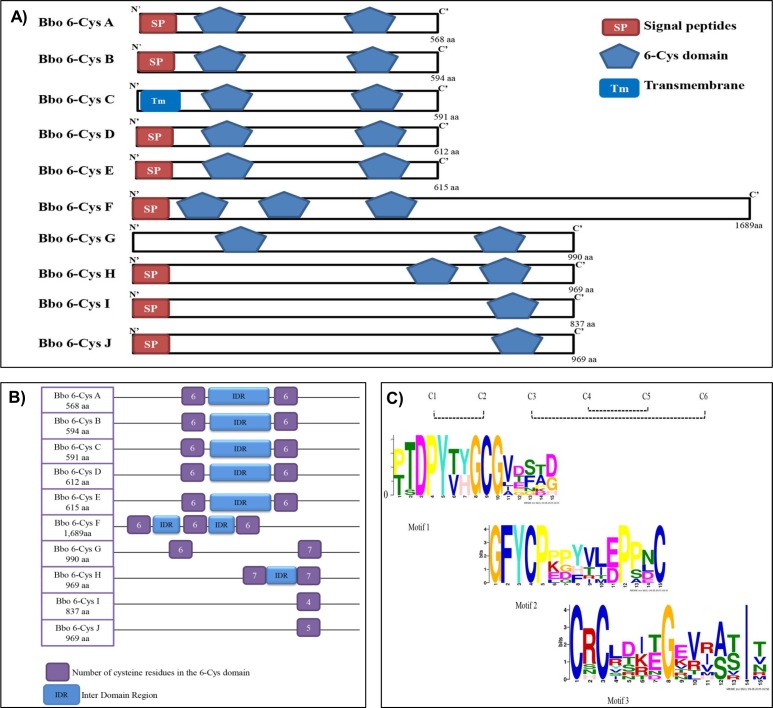
Characterization of Bbo 6-Cys proteins and 6-Cys domain. **A.** Schematic representation of 6-Cys family proteins. The number and relative localization of the 6-Cys domains, represented as blue boxes, the predicted signal peptides (SP) and transmembrane domains (Tm), and the number of amino acids are indicated. **B.** Schematic representation of the arrangement and number of the 6-Cys domains in each of the *B*. *bovis* 6-Cys proteins. The number of residues found in each domain is represented inside each box. The lengths of the inter-domain regions (IDR) in the proteins containing two 6-Cys domains are relatively similar at 163,161,159,162, 164, in the A, B, C, D and E proteins, whereas it contains 227 and 521 aa in H and G’s 6-Cys domain, respectively. Since protein F has three 6-Cys domains so it has two IDRs with lengths 164 aa and 214 aa for IDR1 and IDR2 respectively. **C**. Schematic representation of the predicted disulfide bonds in the *B*. *bovis* 6-Cys proteins using the Prosite program (top). Description of the three conserved domains identified in the 6-Cys domains among the ten 6-Cys *B*. *bovis* proteins using MEME analysis. Amino acids are represented using letter symbols. The highest letters represent strictly conserved residues. Font sizes are proportional to the relative frequency of each residue for each position.

The predicted protein size of the ten Bbo 6-Cys ranges from 568 aa [66.73 KDa] to 1,689 aa [187.27 KDa] (Fig 2A, Table 1). All, except Bbo 6-Cys G, have signal peptide (SP), suggesting that they may be surface exposed or secreted proteins. Earlier gene modeling analysis did not predict a signal peptide for Bbo 6-Cys protein F. Additional visual analysis revealed the presence of a 23 aa hydrophobic SP prior to the three 6-Cys domains (PF07422PF07422-PTZ00360) at the N-terminus of the protein (Fig 2A and 2C, S1 Fig). Bbo 6-Cys protein C is the only member with a predicted transmembrane (Tm) domain in its N-terminus region and none of the Bbo 6-Cys proteins contain glycosyl-phosphatidylinositol anchors (GPI) (Fig 2A). This feature is in contrast to the orthologous Pf48/45 protein which is predicted to contain a GPI anchor and is located on the gamete surface although another orthologous soluble protein, Pfs230, also lacks a GPI anchor [38, 39]

The 6-Cys domain is typically 120 aa long and characterized by the presence of six positionally conserved cysteines (6-Cys) [21]. Although many of the domains in Plasmodium 6-Cys proteins do contain six cysteine (C) residues, there are exceptions in other *Plasmodium* 6-Cys proteins where just four conserved cysteines have been found in the domain [40]. In regards to the Bbo 6-Cys family, there is a single 6-Cys domain in proteins I and J, two in proteins A, B, C, D, E, H and G and three domains in protein F (Fig 2A and 2B, Table 1). The majority of the 6-Cys domains in *B*. *bovis* contains six cysteine residues [A to G domain (D1)], except for D2 of the 6-Cys proteins G and H which contain seven cysteines (Fig 2B). In addition, the single 6-Cys domain in proteins I and J have four and five cysteines, respectively (Fig 2B). *In silico* predictions using “prosite” suggests that, similar to their *Plasmodium* orthologues, the typical 6 cysteine residues of Bbo 6-Cys putative proteins may be involved in disulfide bond formation between C1-C2, C3-C6, and C4-C5 (Fig 2C) [17, 22, 41]

Alignment of the *B*. *bovis* 6-Cys domains shows cysteine residue conservation and 100 percent aa sequence identity between the 6-Cys D2 of protein G and the 6-Cys D2 of protein H (S2 Fig highlighted in yellow). The alignment also demonstrates full conservation of glycine and phenylalanine residues among all Bbo 6-Cys domains. Conservation of glycine and phenylalanine residues is suggested as a requirement for conformational structural preservation through flexibility provided by these residues (S2 Fig). Also of note, there are three additional conserved degenerate motifs among the Bbo 6-Cys protein members (Fig 2C) hereby termed as motif #1[(YH) GCG], motif #2 [GF (YF) CP] and the more degenerate, motif #3 [C(R, S, H) C]. These three degenerate motifs are also conserved in the *Plasmodium* Pf48/45 and Pfs230 proteins [40]. It remains to be determined if these degenerate motifs are widely conserved among all apicomplexan parasites possessing 6-Cys gene family members. Such conservation may imply phylum-wide functions.

Amino acid sequence comparison among all predicted Bbo 6-Cys family proteins with their annotation are shown in Table 3 and their phylogenetic tree is shown in S3 Fig. Both data illustrate the relatedness between the Bbo 6-Cys family members. As shown in Table 3, all 6-Cys proteins are annotated as hypothetical proteins except proteins E and J which are annotated as putative membrane proteins. These comparative sequence analyses which include gene structure, orientation and the number of domains, Bbo 6-Cys protein family can be classified into two subfamilies (SF). The percent of identity among SF-1 ranges from 25% to 53%. The highest identity found (53%) is between proteins A and B, whereas proteins H and G (52.8%) are the most closely related among the SF-2 (Table 3). Alignment among the 6-Cys A and B proteins reveals a fully conserved continuous stretch of 164 aa located at the N-terminus of the proteins which includes a 6-Cys domain (S4 Fig).

**Table 3:** The percent of identity among 6-Cys protein family members.

	**A**	**B**	**C**	**D**	**E**	**F**	**G**	**H**	**I**	**J**
**A**	100	52.81	37.31	39.69	24.96	17.51	19.50	17.33	17.04	17.35
**B**	52.81	100	39.32	31.21	25.69	17.15	18.10	16.64	15.01	16.95
**C**	37.31	39.32	100	32.24	26.08	18.10	18.67	17.93	13.94	16.17
**D**	39.69	31.21	32.24	100	28.52	18.87	19.44	21.02	15.09	15.32
**E**	24.96	25.69	26.08	28.52	100	15.56	18.20	18.01	15.47	15.60
**F**	17.51	17.15	18.10	18.87	15.56	100	37.94	47.29	17.00	21.51
**G**	19.50	18.10	18.67	19.44	18.20	37.94	100	52.80	15.62	21.69
**H**	17.33	16.64	17.93	21.02	18.01	47.29	52.80	100	15.92	22.56
**I**	17.04	15.01	13.94	15.09	15.47	17.00	15.62	15.92	100	16.94
**J**	17.35	16.95	16.17	15.21	15.60	21.51	21.69	22.26	16.94	100

The Bbo 6-Cys subfamily 1 (SF-1) consists of Bbo 6-Cys *A* to *E* encoding protein sizes ranging from 591 to 615 aa in length (average length 602 aa) (Table 1). SF-1 genes are linked, arranged in a head to tail organization and clustered on one side of chromosome 2 (Fig 1). All Bbo 6-Cys subfamily 1 (SF-1) proteins share several similar features including the number and relative localization of sexual stage domains and blocks of conserved sequence motifs (S5 Fig) (Fig 2A and 2B. All SF-1 proteins exclusively contain two typical 6-Cys domains with six cysteines in each domain (Fig 2B). Interestingly, orthologous 6-Cys proteins in *B*. *bigemina*, XP_012766229, XP_012766226 and a membrane protein XP_012766225 have 100% identity within the domain and that the latter two *B*. *bigemina* protein-encoding genes are also tandemly arranged in a head to tail manner on a single chromosome.

Bbo 6-Cys subfamily 2 (SF-2) includes genes *F* to *J*. Proteins G and H contain two 6-Cys domains while proteins I and J contain a single domain (PTZ00360) whereas protein F has three domains (Fig 1). In contrast to the SF-1 proteins, the majority of these domains in SF-2 are located exclusively in the C-terminus, except for proteins F and G which contain 6-Cys domain at the N-terminus (Fig 2A and 2B). SF-2 proteins have a conserved motif (S6 Fig) that is also found in 6-Cys proteins in *B*. *bigemina*, *T*. *parva and T*. *annulata*.

### Transcriptional analysis of Bbo 6-Cys via microarray, RNA seq and RT-PCR

**Fig 3:**
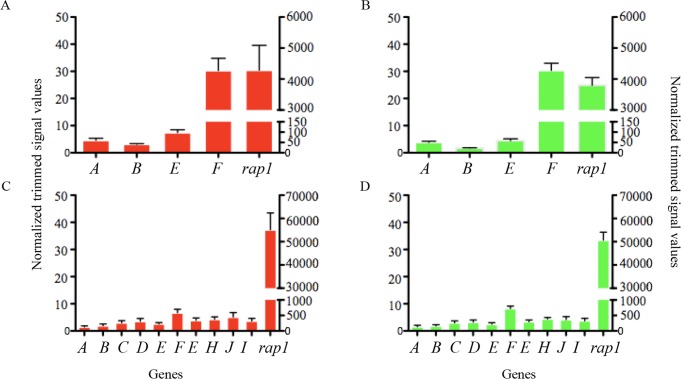
Transcriptional analysis of the Bbo 6-Cys genes in cultured blood stages. **A. and B.** RNA seq analysis performed on the virulent (red) and attenuated (blue) parasites derived from the L17 strain of *B*. *bovis*. The Y axis indicates relative transcriptional levels. The X axis represents the name of the 6-Cys and rap-1 control genes. Transcripts for the *B*. *bovis* 6-Cys genes *C*, *D*, *G*, *H*, *I* and *J* were not detected using RNA seq. **C. and D.** Microarray analysis of the virulent (red) and attenuated (blue) parasites derived from the T2Bo strain of *B*. *bovis*. The Y axis indicates relative transcriptional levels. The X axis represents the name of the 6-Cys and *rap-1* control genes.

**Fig 4:**
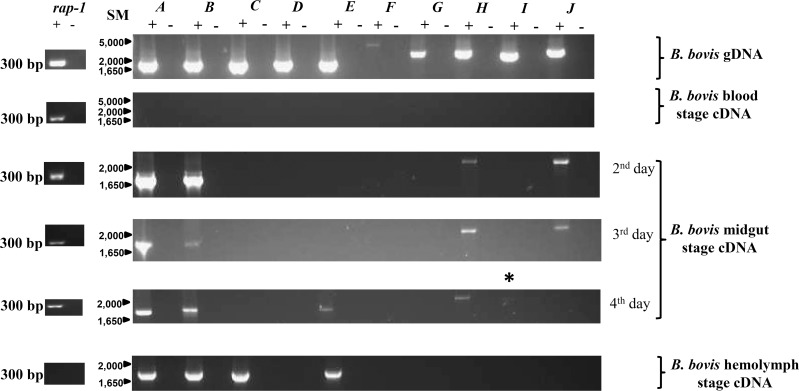
Differential transcription of the Bbo 6-Cys family gene members among three distinct developmental stages of the life cycle of *B*. *bovis*. **First panel:** PCR amplifications of the full ORF of each Bbo 6-Cys family gene members (*A-J*) using gDNA extracted from cultured *B*. *bovis* T3bo strain parasites. Amplifications were performed on samples with (+) or without (-) DNA template. PCR amplification of the *B*. *bovis* RAP-1 gene was used as a control (left panel). **Second panel:** Transcription analysis of the Bbo 6-Cys family gene members(*A-J*) using cDNA generated from total RNA extracted from *in vitro* blood culture T3bo strain parasites. Identical results were obtained using RNA extracted from *in vitro* cultured Mo7 clonal line, L17 strain; *in vivo* T3Bo strain and T strain pair virulent and attenuated (data not shown). Identical amplifications using RAP-1 primers generated a 300 bp PCR product (indicated in the left panel). **Third panel:** Transcription analysis of the Bbo 6-Cys family gene members (*A-J*) performed on cDNA generated from total RNA extracted from *B*. *bovis*-infected *R*. *microplus* midgut. Total RNA was extracted from midguts at different days upon incubation (2, 3 and 4 day) at 28°C. Asterisk (*) indicate presence of faint band for 6-Cys *I* gene amplification. Amplification of the control rap-1 gene is shown in the left panel. **Fourth panel:** Transcription analysis of the Bbo 6-Cys family gene members (*A-J*) using cDNA generated from total RNA extracted from *B*. *bovis* infected *R*. *microplus* hemolymph. Control gene RAP-1 was not amplified from infected hemolymph. Size markers (SM) are indicated on the left of the large panels.

Transcriptome analyses using short-term cultured merozoites from strains differing in origin and virulence show that the members of the Bbo 6-Cys family are either transcribed at low or undetectable levels compared to constitutively expressed *rap1* in the blood stages. Transcriptional RNA sequence analysis performed using virulent and attenuated *B*. *bovis* L17 parasites (Fig 3A and 3B) ([42]; www.piroplasmadb.org) indicates that transcription of Bbo 6-Cys genes *C*, *D*, *G*, *H*, *I* and *J* were not detected while genes *A*, *B*, *E* and *F* were transcribed at relatively lower levels than *rap-1*[42]*]* (Fig 3A and 3B). Transcription of Bbo 6-Cys *F* was the most abundant in comparison to the rest of the gene family members (Fig 3A and 3B). Concurrent microarray analysis of the virulent and attenuated T2Bo *B*. *bovis* parasites substantiates the low transcriptional levels of all 6-Cys family members in comparison to *rap-1* by RT-PCR (Fig 3C and 3D). Transcriptional analyses using various strains and sources of *B*. *bovis* blood stage parasites [i) cultured blood stage parasites from *B*. *bovis* T3Bo, culture Mo7 clonal line, Argentina L17 (virulent), and two Australian (T) strains (virulent and attenuated) [31], (ii) blood of the experimentally infected animal with *B*. *bovis* by RT-PCR is shown in Fig 4. Although *rap-1* transcription was detected, none of the 6-Cys transcripts were amplified (Fig 4 second panel) within blood stages.

However, the temporal detection of some Bbo 6-Cys gene transcripts within the tick midgut (Fig 4 third panel) suggests differential regulation. Genes *A*, *B* and *H* are constitutively expressed throughout day 2 to 4 post-tick feeding (ptf) within the midgut (Fig 4, third panel). Gene *J* is transcribed inside the tick midgut on days 2 and 3 ptf but its transcript is not detected on day 4 ptf (Fig 4, third panel). On day 4 ptf gene *J* is no longer detectable, but RT-PCR also detected transcripts of the genes *E* and *I* (Fig 4, third panel). Transcriptional analysis in the motile kinete stage within hemolymph reveals the expression of genes *A*, *B*, *C* and *E* (Fig 4 fourth panel). Transcription of rap*-1* was detected in the blood and infected tick midgut stages (Fig 4 fourth panel) but not in infected hemolymph. Transcripts of *B*. *bovis* 6-Cysgenes were not detected in identical RT-PCR performed on total RNA extracts from uninfected *R*. *microplus* midgut (data not shown).

### The Bbo 6-Cys A and B are translated by *B*. *bovis* parasites in the *R*. *microplus* midgut

**Fig 5:**
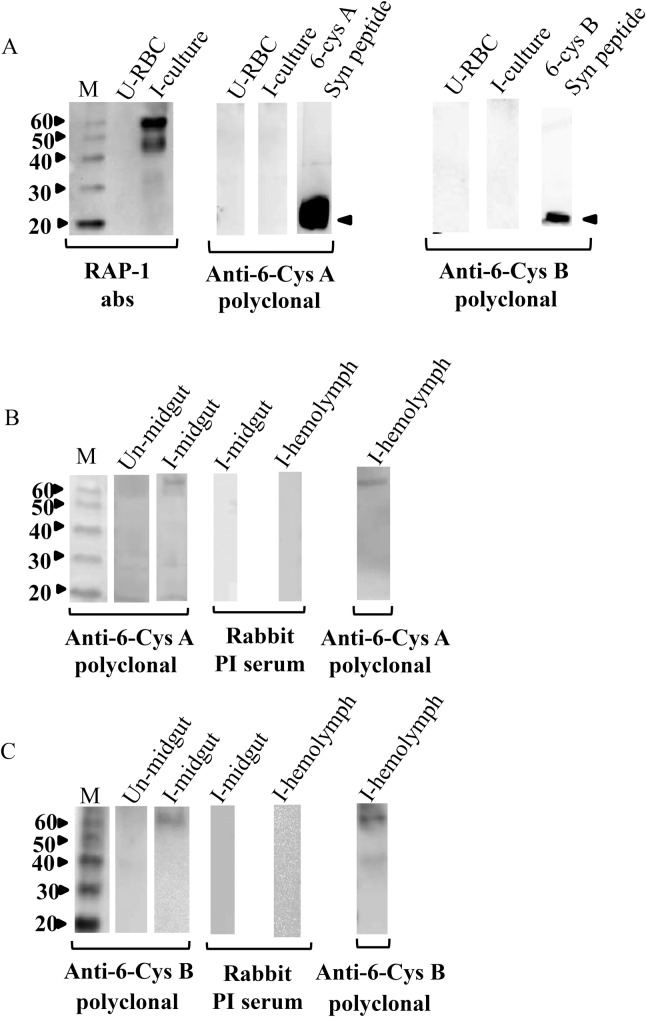
**Immunoblot analysis for expression of the *B*. *bovis* 6-Cys A and B proteins in blood, midgut, and hemolymph stages**. **A.** Immunoblot analysis of uninfected RBC and cultured *B*. *bovis* infected RBC with rap-1 mAb BABB75, rabbit polyclonal sera against 6-Cys A-specific synthetic peptide and 6-Cys B-specific synthetic peptide. Synthetic A and B peptides were used as positive controls indicated by the black arrow. The size of peptide A is 21.6 D and peptide B is 24 D. **B.** Immunoblot analysis of non-infected and *B*. *bovis* infected *R*. *microplus* midgut protein extract and hemolymph with rabbit polyclonal sera against 6-Cys A-specific synthetic peptide, and pre-immune rabbit sera (PI). **C.** Immunoblot analysis of non-infected and *B*. *bovis*-infected *R*. *microplus* midgut protein extract and hemolymph with rabbit polyclonal sera against 6-Cys B-specific synthetic peptide, and pre-immune rabbit sera. Size markers (M) in kDa are indicated at the left of each panel.

Transcriptional data demonstrate that Bbo 6-Cys genes *A* and *B* are constitutively transcribed in tick midgut and hemolymph during the parasite’s life cycle. To investigate if the Bbo 6-Cys genes A and B are translated in these stages, monospecific polyclonal antibodies against peptide cocktails derived from both proteins were used in western blot analysis. RAP-1 monoclonal antibodies were used as a positive and negative control in these western analyses and as expected, RAP-1 was detected infected blood but not hemolymph. Synthetic peptides specific to proteins A and B were also included as controls in the western blots and as expected, reacted positively with antibodies against peptide cocktails specific to proteins A and B (Fig 5A). In contrast, the Bbo 6-Cys proteins A and B were not detected in cultured red blood cells infected with *B*. *bovis* in identical immunoblots (Fig 5A). However, the 6-Cys A and B proteins were detected in both midgut and hemolymph stages of *B*. *bovis*. Faint bands of approximately 66 kDa which correspond to both proteins A and B were observed in the immunoblots (Fig 5B and 5C). Collectively, these data support the differential expression of the 6-Cys A and B proteins in midgut and hemolymph stages of the parasite.

## Discussion

Production of safe and efficacious vaccines to control babesiosis and related malaria has eluded biomedical research. The ability of causal apicomplexan parasites to evade and persist in the presence of host immune responses is a primary challenge in vaccine development. The search for immune vulnerable events in the complex life cycles of *Babesia* and malarial parasites led to exploration of sexual reproduction within their arthropod vectors. Among the many similarities between *P*. *falciparum* and *B*. *bovis*, both parasites undergo sexual reproduction in the gut of its respective vectors although molecular mechanisms of this process remain largely uncharacterized especially in *B*. *bovis*.

Fourteen 6-Cys domain-containing genes have been identified in *Plasmodium* [40] and all of them are predicted to encode surface exposed proteins that are differentially expressed in the parasite’s life cycle in stage-specific manner [21]. Here we demonstrate that the *B*. *bovis* genome contains at least ten 6-Cys genes, rather than the six that were previously described [24]. Remarkably, there is a dramatic expansion of this gene family in *B*. *bigemina*, and thus the significance of this observation should be further investigated. This dramatic expansion may contribute to the genome size differences (*B*. *bigemina* 13.84 Mb [43]) versus (*B*. *bovis* 8.2 Mb [32]) which may ultimately explain species-specific genes and distinctive gene duplication of conserved gene families [43]. As studies show that the 6-Cys gene family members of *Plasmodium* parasites are differentially expressed throughout the life cycle, data here demonstrate that the *B*. *bovis* 6-Cys genes are predominantly expressed during parasite replication in the tick. These differences between *Babesia* and *Plasmodium* may ultimately reflect divergence in life cycles and transmission vectors. After all, *Plasmodium* sporozoites must invade liver cells prior to the establishment of asexual blood stages while *Babesia* sporozoites invade erythrocytes directly; *Plasmodia* gametocytes develop inside host erythrocytes while all evidence suggests that the sexual stages of *Babesia* occurs only within the vector.

The 6-Cys families of proteins appear to be Apicomplexan specific and are characterized by containing conserved arrangements of 6-Cys protein residues defining domains of near 120 aa in size. However, this definition was recently revised upon the description of several 6-Cys proteins lacking the typical arrangement of the watermark 6-Cys protein residues characteristic of the s48/45 domain, but instead, an alternative domain defined by just four cysteine residues [40]. A typical example of such proteins is the *P*. *berghei* Pb48/45 containing three 6-Cys domains, with the first and the second domains having an arrangement of four cysteine residues [17]. This is also true for other *Plasmodium* 6-Cys-related proteins such as sequestrin (PF3D7_04050300) and Pf92 (PF3D7_1364100) [44], the first 6-Cys domain of Pfs47, as well as other 6-Cys domains present in Pfs230 and Pfs230p [17]. Furthermore, s48/45 domains can also be defined by the presence of five cysteine residues, as it was found for 6-Cys domains identified in Pf48/45 and Pfs38 6-Cys proteins[17]. Collectively, these data support that the Pf48/45 domains of *Plasmodium* parasites can, in fact, be detected more accurately by extending the definition to the conservation of just four, five, or six positional conserved cysteine residues [40]. The analysis of the 6-Cys domains of the *B*. *bovis* 6-Cys proteins shown in this study suggests that the 6-Cys domains of this parasite may uniquely contain, “6-Cys” domains containing up to seven positionally conserved cysteine residues, in addition to the previously described *Plasmodium* “6-Cys” domains composed of 4 and 5 residues.

We previously described the transcription of Bbo 6-Cys gene *E* in the merozoite stages of the *B*. *bovis* Mo7 strain [24]. In this study, we were not able to confirm gene *E* transcription by RT-PCR, as well as the other Bbo 6-Cys genes in erythrocyte stages of *B*. *bovis*. The results in this study corroborate with those from the transcriptome analysis previously reported by Pedroni et al.[42] using two very different *B*. *bovis* strains. Yet, the previous expression analysis was performed solely using *in vitro* cultured Mo7 parasites. An important difference precluding direct comparisons among these strains is the clonal nature of the Mo7 strain compared to the polyclonal structure of the T3B strain, and the changed conditions using in the *in vitro* culturing adopted by our lab after performing these previous studies. These changes could result in ambiance differences that might have affected dramatically the pattern of expression of the 6-Cys genes. It remains to be confirmed whether the T3B strain can also express the 6-Cys *E* gene in blood stages growing under distinct parasite developmental conditions.

We propose a future revision on the annotation for the Bbo 6-Cys *F* gene. First, it is very likely that nucleotide sequence located immediately 5’ of the initial start of the *F* gene, is responsible for the coding of a previously unannotated 25 aa hydrophobic peptide sequence that might well represent a possible signal peptide for the protein F. Secondly, the annotated version of the 6-Cys RNA (GB accession numberXM_001609595) predicted an ORF of 5001 bp encoding for a protein of 1,660 amino acids. Although supported by the presence of the canonical splicing sites on the putative intron/exon boundaries [45], this gene model results in a putative 6-Cys protein that differs dramatically with the other members of the *B*. *bovis* 6-Cys family, and was deduced from a gene structure containing 9 exons and 8 introns. However, the first exon encodes for a 2,859 bp ORF that encodes for a 953 aa protein including three 6-Cys domains. This large exon is followed by a 300 bp intron, which might be considered large in size compared to the other introns present in this genomic region (typically 38–91 bp long). In our attempts for detecting 6-Cys *F* transcripts in erythrocyte stages using RT-PCR, we found that although the sequence representing the large exon 1 of the ORF was not amplifiable, we were able to amplify the 3-terminus region of the transcript representing regions encoded by the exons 7 and 8 of the gene (data not shown). Although inconclusive, this observation is indicative of the actual presence of at least two distinct genes encoded in the 5,707bp genomic region including the annotated 6-Cys gene *F*. While re-annotation of the *B*. *bovis* 6-Cys genes is beyond the scope for this study, it is possible that an alternative definitive gene model can be generated for this gene if more information can be gathered from transcriptional RACE and RT-PCR analysis performed on RNA derived from the stages were the 6-Cys *F* gene is abundantly expressed.

Sequence divergence analysis of 6-Cys family members comparing the sequence of each 6-Cys gene among five geographically distinct *B*. *bovis* strains shows that the average of the ω (dN/dS) ratio for all *B*. *bovis* 6-Cys family members are, in all cases, < 1 indicating that all of these genes are under purifying selection pressure. This includes the untypical gene F, and so if the F gene is re-annotated, the dN/dS ratio should be recalculated. However it is unlikely that recalculation results in a different conclusion than the one arising from our analysis since, and as reflected by a ratio that is lower than 1, the whole region used in our calculation appear well conserved among all the strains analyzed. This finding implies the lack of exposure of the 6-Cys proteins to the immune system, which is in agreement with the lack of expression of these genes during the erythrocyte stages of the parasite. The high level of sequence conservation among distinct *B*. *bovis* strains and the lack of expression in the erythrocytic stages are two attractive properties of this protein family as potential vaccine candidates, since they may be useful at inducing transmission blocking antibodies even when animals are challenged with heterologous strains by interfering with parasite reproduction in tick. On the other hand, the limited sequence polymorphisms found among the 6-Cys proteins may indicate that strict conservation is needed in order to perform the biological functions of these proteins. Overall, the data may indicate roles of the 6-Cys family gene members during the parasite life cycle inside the tick, including presumably, the apicomplexan-conserved mechanism involving the development of sexual stage (gamete) parasites. In the *Plasmodium* parasites, transcripts of the 6-Cys genes that are associated with sexual stage development can readily be detected in cells developing inside their vertebrate host, signaling commitment of some parasites to the sexual pathway. In contrast, data shown here suggest that most of *B*. *bovis* 6-Cys family members are differentially expressed in life stages of the parasites occurring outside the vertebrate host, an observation which aligns well with the previously recognized lack of sexual stages in the blood stages of *B*. *bovis*.

The findings presented here indicate Bbo 6-Cys *A* and *B* proteins are of particular interest as potential targets for the development of TBV. These two highly related proteins are almost fully conserved among geographically distinct strains and have a similar transcription pattern among different developmental stages of *B*. *bovis*, including high levels of expression in the early stages of gamete development within the tick midgut and kinete. Their sequence similarities, same domain distribution pattern in their protein and close contiguous localization in the genome suggest that the 6-Cys *A* and *B* genes are paraloguous genes originated from the duplication of a single ancestral gene. Collectively, these data suggest that the 6-Cys proteins A and B are markers for *B*. *bovis* sexual stage and the strongest 6-Cys family member candidates thus far identified for the development of a *B*. *bovis* TBV. Importantly, previous work in *Plasmodium* demonstrated that antibody effectors against at least two 6-Cys proteins, P48/45 and P230, [21, 46, 47] are able to block the development of *Plasmodium* sexual stages and thus, inhibit sexual reproduction.

In summary, the data presented here along with the body of evidence so far accumulated in the *Plasmodium* system, validate the approach of developing *Babesia* transmission blocking 6-Cys-based vaccines. Because 6-Cys genes *A* and *B* are highly conserved, expressed early in the process of sexual stage cell development, encode for putative surface proteins and their sharing characteristics with the leading TBV vaccine candidates in *Plasmodium*, they are markers for *B*. *bovis* sexual stage development in the tick midgut, and strong initial candidates for developing TBV against *B*. *bovis*. Ongoing experiments are currently being developed to demonstrate the functional significance of these two genes, as well as their potential as components of a *B*. *bovis* TBV.

## Supporting Information

S1 Fig *B*. *bovis* 6-Cys F gene encoding protein criteria. A) Schematic representation for the Putative Signal Peptide (SP) for 6-cys F found in are upstream for the original annotated protein CDS (coding sequence) in *B*. *bovis* genome.B) Diagram representing the 9 exons of 6-Cys *F* gene with the length of the 8 introns between the exons. The biggest exon is 2859bp while the smallest one is 66bp.
(TIF)

S2 Fig Sequence alignments of the 6-Cys domains from the Bbo 6-Cys proteins using Clustal omega. The conserved 6-Cys cysteine residues among the *B*. *bovis* 6-Cys domains are marked with a blue box, and other positionally conserved amino acids are pointed out by red arrows. The identical domains G6-Cys2 and H6-Cys2 are marked with a yellow shade. Asterisks (*) indicate fully conserved residues, (:) indicates amino acid conservation between groups of strongly similar properties, (.) Indicates conservation between groups of weakly similar properties.
(PDF)

S3 Fig Phylogenetic tree among all members of Bbo 6-Cys family. The phylogentic analysis generated by the phylogeny.fr. The blue color shade subfamily 6-Cys #1 and the red color shade the subfamily 6-Cys #2.
(TIF)

S4 Fig **Complete sequence alignments of the 6-Cys A and B proteins.** The alignment shows the fully conserved continuous stretch of 164 aa located at the N-terminus of the proteins which includes part from the first 6-Cys domain in both protein. The black arrows indicate the conserved cysteine residues in both proteins. The red lines indicate the borders of 6-Cys domains.
(PDF)

S5 Fig Sequence alignments of the full 6-Cys proteins of the subfamily 1(SF1). A conserved motif identified in the 6-Cys SF1 proteins is pointed out by red box. Residues depicted in white font over black background indicate conserved amino acids.
(PDF)

S6 Fig Sequence alignments of the full 6-Cys proteins of the subfamily 2(SF2). A partially conserved motif identified in the 6-Cys SF2 proteins is pointed out by red box. Residues depicted in white font over black background indicate conserved amino acids.
(PDF)

S1 Table Primers for gene amplification. (DOCX)

S2 Table Primes for sequencing. (DOCX)

S3 Table Genbank accession numbers for gene members of the Bbo 6-Cys family in five different strains. Bbo 6-Cys genes from A to J in the Tx attenuated strain, T-virulent and attenuated strains, L17, and Mo7 clonal line.
(DOCX)

S4 Table Amino acid sequences of the synthetic peptides used for the production of rabbit polyclonal antibodies against the Bbo6-Cys proteins A and B. More than one synthetic peptide was used as a cocktail for the production of antibodies against the proteins. The optical density [OD] values obtained in ELISA reaction testings’ for each of the peptide cocktails using rabbit sera 8 weeks after the start of the immunizations (1:200 dilution) is shown in the table.
(DOCX)

S5 Table Genes containing s48/45 domains in: B. bigemina [Bond strain], *B*. *microti* [R1], *Theileria equi* [WA strain], *T*. *parva* [Muguga strain] and *T*. *annulata* [Ankara strain]. (DOCX)
