# Supplementary figures and images for: Expression of 6-Cys Gene Superfamily Defines Babesia bovis Sexual Stage Development within Rhipicephalus microplus
Source: PLoS One. 2016 Sep 26;11(9):e0163791. doi: 10.1371/journal.pone.0163791 (PMC5036836; doi:10.1371/journal.pone.0163791)

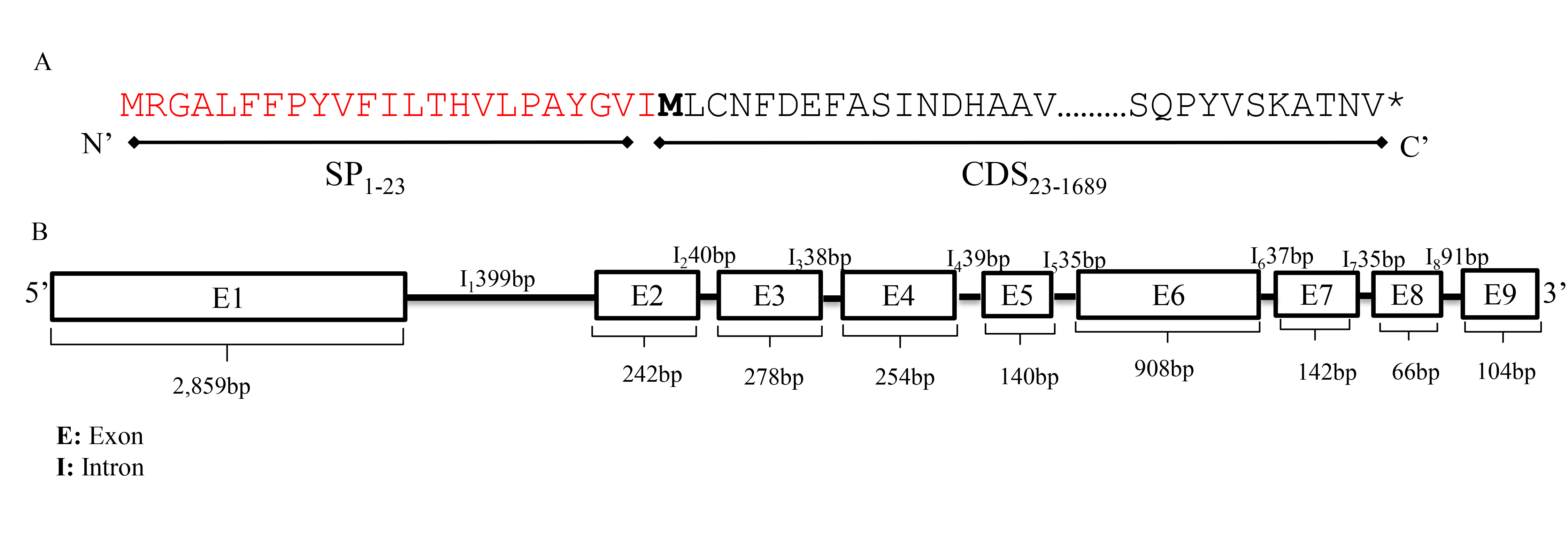

Supplement: S1 Fig — A) Schematic representation for the Putative Signal Peptide (SP) for 6-cys F found in are upstream for the original annotated protein CDS (coding sequence) in B. bovis genome.B) Diagram representing the 9 exons of 6-Cys F gene with the length of the 8 introns between the exons. The biggest exon is 2859bp while the smallest one is 66bp. (TIF) [file pone.0163791.s001.tif]

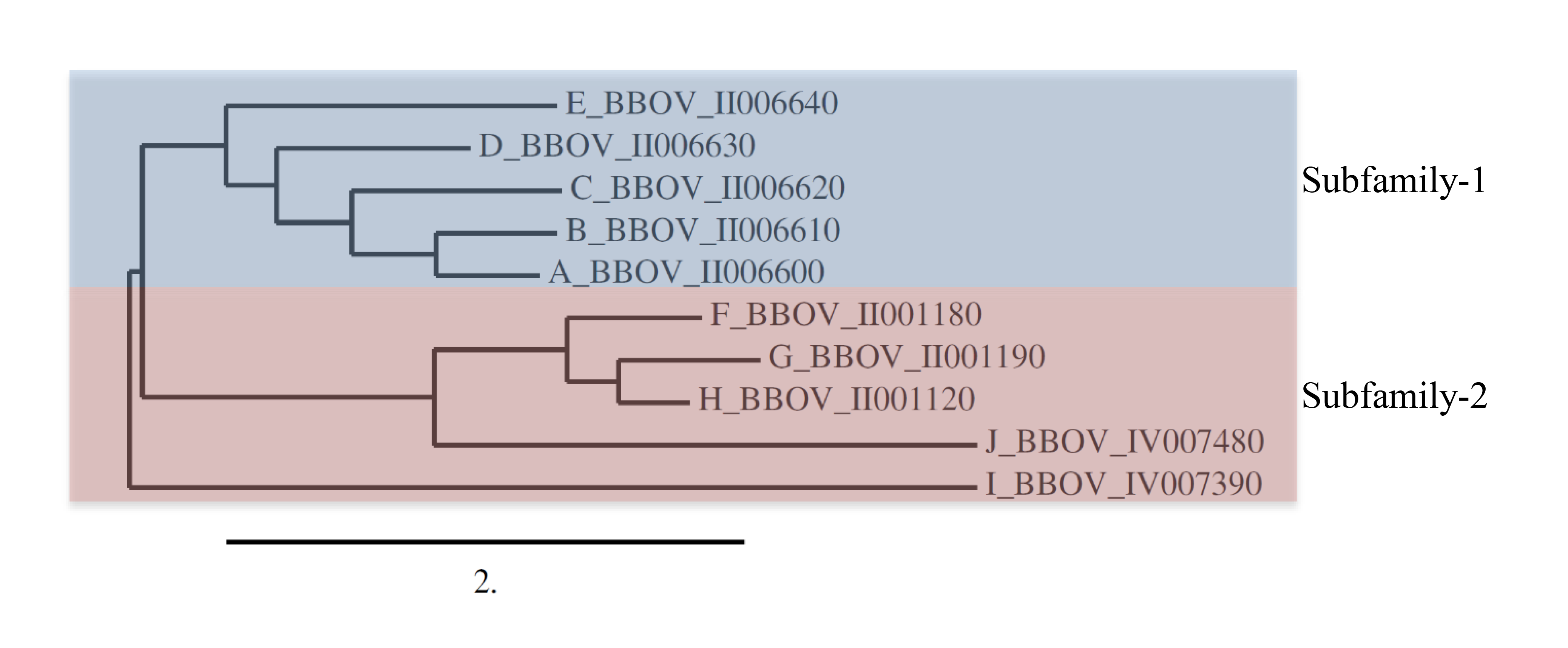

Supplement: S3 Fig — The phylogentic analysis generated by the phylogeny.fr. The blue color shade subfamily 6-Cys #1 and the red color shade the subfamily 6-Cys #2. (TIF) [file pone.0163791.s003.tif]
