# Supplementary material for: Expression of 6-Cys Gene Superfamily Defines Babesia bovis Sexual Stage Development within Rhipicephalus microplus
Source: PLoS One. 2016 Sep 26;11(9):e0163791. doi: 10.1371/journal.pone.0163791 (PMC5036836; doi:10.1371/journal.pone.0163791)
Supplement: S2 Fig — The conserved 6-Cys cysteine residues among the B. bovis 6-Cys domains are marked with a blue box, and other positionally conserved amino acids are pointed out by red arrows. The identical domains G6-Cys2 and H6-Cys2 are marked with a yellow shade. Asterisks (*) indicate fully conserved residues, (:) indicates amino acid conservation between groups of strongly similar properties, (.) Indicates conservation between groups of weakly similar properties. (PDF) [file pone.0163791.s002.pdf]

```
I6cys     -------GKTDPSELKSVQLQDEFAVVGISMYKKPEVNLGVDLEFSFEADSKIWAVHKKP
E6cys2    ------------------------------------------------------------
A6cys2    ------------------------------------------------------------
D6cys2    ------------------------------------------------------------
B6cys2    ------------------------------------------------------------
C6cys2    ------------------------------------------------------------
F6cys1    ------------------------------------------------------------
G6cys1    ------------------------------------------------------------
J6cys     ITFNFICGKVSPGLDNADKVE-K--YSGRFRYTNPIH--------REDRLMEIWGLIK--
F6cys3    ------------------------------------------------------------
G6cys2    ------------------------------------------------------------
H6cys2    ------------------------------------------------------------
F6cys2    ------------------------------------------------------------
H6cys1    ------------------------------------------------------------
E6cys1    ------------------------------------------------------------
D6cys1    ------------------------------------------------------------
B6cys1    ------------------------------------------------------------
A6cys1    ------------------------------------------------------------
C6cys1    ------------------------------------------------------------

I6cys     IYFVCAKKGYKHGQNSHAYIAFDPLYQLGRLYGCG--TR-PELFLNEEGQ----------
E6cys2    -----------------------------YTYGCG--AENPDIFNTKGVV----------
A6cys2    -----------------------------YTYGCG--VDSADLFRDTGFQ----------
D6cys2    -----------------------------YTYGCG--VDSADLFHKEGFL----------
B6cys2    -----------------------------YTYGCG--VDSADLFRKDGFK----------
C6cys2    -----------------------------YTYGCG--VESTDLFRTDGFQ----------
F6cys1    --------------------------------GCG--SRSSSLFLDKQLV----------
G6cys1    --------------------------------GCG--SRASPLFRNPNDV----------
J6cys     ---------------------ITIPTTDPYVHGCGIPGDHDKLFLPDTDI----------
F6cys3    -----------------------------YVHGCGLGFKGETLFRDDTIT----------
G6cys2    -----------------------------YVHGCGVTFTGEEIFKPDTVH----------
H6cys2    -----------------------------YVHGCGVTFTGEEIFKPDTVH----------
F6cys2    -----------------------------DTTGCD--IGQPSVFNPRNMAKYIRPKEYIG
H6cys1    -----------------------------DIKGCD--SHQTPIFDPSLVAKDCVWKKYEY
E6cys1    -----------------------------IPYGCG--SAGYHMFKNSIPI----------
D6cys1    -----------------------------MAYGCE--SSDSALFLNGIPQ----------
B6cys1    -----------------------------MAYGCE--TGNNMLFKNTIPK----------
A6cys1    -----------------------------MAYGCE--TGNNMLFKNSRPI----------
C6cys1    -----------------------------MAYGCE--TGNNMLFKNTAPF----------
                                          **        :*

I6cys     RN-----------SNTHCVFKIDD-RKTVGFFCPSP------------------------
E6cys2    FNNQHIQKGAHYHTEVKCTLNAWK-NSPIGFYCPKQYVLEPADCFNSAYLVSTNHVVRLD
A6cys2    LKQEGR-----GRKVTHCKVNPYL-SSPVGFYCPEGFVLEPPNCFSEMLHKDKEVVVPLS
D6cys2    LSFEYD-----QVPVTKCKVNPYL-SSPVGFYCPEGFVLEPPNCFSEMLHKDKEVVVPLS
B6cys2    LSTNTE-----DEDVTECKVNPYL-TSPVGFYCPKDHTLEPSNCFEEMINATNNEKVKLS
C6cys2    ISKQEE-----HVSITKCKINPYL-TSPVGFYCPKDHTLEPSNCFEEMINATNNEKVKLS
F6cys1    DVDADT-------GVRSCEVDPMS-STPIGFLCEG--RMEPPECMKYLI--DTNGKIRPN
G6cys1    IIDEAT-------GLRTCVVDPMS-TLPIGFLCEG--EIEPPDCFRYLI--SDNNYVHHP
J6cys     LRDRAG-------KIIGCSVNMKR-AGRAGFYCPLPYRTDPPDC----------------
F6cys3    YTEPTS-------GIKSCVIDLNV-NDEGGFYCPPPYIMEPPECFHDVF--VGDVVTKLN
G6cys2    ITDAND-------GSSGCQVDLSE-HRECGFYCPPPYVIDPPLCFQEVL--VDGVVTQLS
H6cys2    ITDAND-------GSSGCQVDLSE-HRECGFYCPPPYVIDPPLCFQEVL--VDGVVTQLS
F6cys2    NTGLMH---D-------CVVKNFFGVFHTGIYCGSGNTLMPNGCKQTAYSLYSNTEEPIP
H6cys1    INGFLH---K-------CVLENAYGAIKAGIFCKDGESLMPNNCLENLYDYSLGKTTRLP
E6cys1    GPDTPH---DKVLEAAQCEIEAEP-NMILGIYCSKDDYVYPDDCFRQVIGMD-GKRIMF-
D6cys1    EDESIS---IKR-QRRLCYIDPIP-NMIIGIYCKPGDHLYPSRCFQEAVLDAQGFTIRFN
B6cys1    LDWLNH---DD--VEEECLITLEP-NMLFGIFCEKGERIWPKNCINDDDYKKMNHAISSY
A6cys1    IPNIL-----NL-QIASCSVDIEP-GMIFGIYCKAGERLDPGECFSDEELSDYNGAITPY
C6cys1    VPGFKV---SDV-DRVDCSLTLEP-GMIFGIYCKPGEQLMPDHCFPGKRLQELNGDITPY
                           * .         *: *
```

```
I6cys         ------------------------------------------------------------
E6cys2        EYAPQG-----RVLNSPNLRIIDFTGPKSVKYTKKYLEESLQCRCRRRD-----------
A6cys2        DFEPLA-----RALEGRHIKVADFHTSTSNRDHIRYSSVELMCRCLDKE-----------
D6cys2        DFEPLA-----RALEGRHIKVADFHTSTSNRDHIRYSSVELMCRCLDRE-----------
B6cys2        DFAPLA-----RTVESKNIRIVDFNLPKKMKKEIIYNKDMLSCRCRDKN-----------
C6cys2        DFAPFA-----RSVESTNLKVADFHMSPRMKKDTKYTDTELKCSCIDKE-----------
F6cys1        RTERWT-----LMNRSTLVVAQPLTYLA---------TSLFEGHCLCIDPLTDRVLAKIV
G6cys1        LVHRYM-----TAYNDTLLIVQPFVYLA---------SPLIEGYCVCRDR----------
J6cys         ------------------------------------------------------------
F6cys3        RISSDL-----FAFRSSHFSLIRLDRLANNAKESRKTYPALRCRCVTIT-----------
G6cys2        DISDSM-----VYHKSTHFSLLSLHHSHKQSNQEPKMSPPLQCRCLSIT-----------
H6cys2        DISDSM-----VYHKSTHFSLLSLHHSHKQSNQEPKMSPPLQCRCLSIT-----------
F6cys2        SSVVRL-----DLLHVEGMQMFKILT------N-TETIFSMSCNCVDPRGFVISRLILEN
H6cys1        QSVKSL-----TYAKVSGMRILQLSY------N-NTRPLSVGCSCVDSQ-----------
E6cys1        NQF-R-EYSFPHNLIDGNMRLMKLSP------KAFNHDMSFSCQCRNKD-----------
D6cys1        KSYIDP--DFPYQEFPERFRLIKLSH------D-FDKKAPISCSCVDRN-----------
B6cys1        SLPWSPTVYRSMSRLSPRFKLFQVSP------NEIVKNINIHCYCQNKE-----------
A6cys1        IPKFAA-NANPASTLSTRFRLFKVHD------GQLPNSVDLSCACVGAY-----------
C6cys1        ANETEF-Y-DSRRKINSRFQLFVVSD------KGVRTHFYHHCYCQGPQ-----------
```
