# Supplementary material for: Expression of 6-Cys Gene Superfamily Defines Babesia bovis Sexual Stage Development within Rhipicephalus microplus
Source: PLoS One. 2016 Sep 26;11(9):e0163791. doi: 10.1371/journal.pone.0163791 (PMC5036836; doi:10.1371/journal.pone.0163791)
Supplement: S4 Fig — Complete sequence alignments of the 6-Cys A and B proteins. The alignment shows the fully conserved continuous stretch of 164 aa located at the N-terminus of the proteins which includes part from the first 6-Cys domain in both protein. The black arrows indicate the conserved cysteine residues in both proteins. The red lines indicate the borders of 6-Cys domains. (PDF) [file pone.0163791.s004.pdf]

```
(A)BBOV_II006600   1 MDIQNTLNRLALACFIV-FCKNLLIATATPSIHLDLSHNNKQYDDFSVNEVIVAGPKEVV
(B)BBOV_II006610   1 MSQLNLLNIFSITFLSVFLTTSSLCSTATPSIHLDLSHNNKQYDDFSVNEVIVAGPKEVV
consensus          1 *   * **   .    *.     *  **********************************

(A)BBOV_II006600  60 ITCGNGRDEDVEHTMYPSDPVSKMLLPPEGNDFTSAVEKEVASHSFYRSSDLNFEVKKAK
(B)BBOV_II006610  61 ITCGNGRDEDVEHTMYPSDPVSKMLLPPEGNDFTSAVEKEVASHSFYRSSDLNFEVKKAK
consensus         61 ************************************************************

(A)BBOV_II006600 120 DVPVSVKISRTVDTLIMAKDPENFSLNFACKYQSKDGSNAPVYKWVTIKFEAVYPMAYGC
(B)BBOV_II006610 121 DVPVSVKISRTVDTLIMAKDPENFSLNFACKYQSKDGSNAPVYKWVTIKFEAVYPMAYGC
consensus        121 ************************************************************

(A)BBOV_II006600 180 ETGNNMLFKNSRPIIPNILNL-QIASCSVDIEPGMIFGIYCKAGERLDPGECFSDEELSD
(B)BBOV_II006610 181 ETGNNMLFKNTIPKLDWLNHDDVEEECLITLEPNMLFGIFCEKGERIWPKNCINDDDYKK
consensus        181 **********. * .  .   .    * . .** *.***.*  ***. *  *  *..   

(A)BBOV_II006600 239 YNGAITPYIPKFAAN-ANPASTLSTRFRLFKVHDGQLPNSVDLSCACVGAYDKTTRILYV
(B)BBOV_II006610 241 MNHAISSYSLPWSPTVYRSMSRLSPRFKLFQVSPNEIVKNINIHCYCQNKERITRKITIK
consensus        241  * **. *   .   .    * ** **.** *    .   . . * *       * .*  

(A)BBOV_II006600 298 NMLGNVVVNASK--QTNNMKADLMPTIKEVIMNMNPGNNITVKYVPDGEVFLSRGRKVTG
(B)BBOV_II006610 301 KLVNKSIDFYRLLSTADKNDAS----VFHRMEYLEPGSKNGFNIPLSGTFKLGSFGMVSG
consensus        301  ..   .     ..      * .....   .  . **          *   *     *.*

(A)BBOV_II006600 356 QLRPTNPDTTAFNGLPVESNPPTPIKDFIGSRGVDITYTKVKDKMVYKVKYADDALLVLK
(B)BBOV_II006610 357 RIFPSNLTYNAYYSLGSSPFVLSRLRQFIGYDGVGISYTKYKGKMKYTFNYKKDSTLVAK
consensus        361  . *.*    *.  *       . ..  ***  ** *.*** * ** *   *  *  ** *

(A)BBOV_II006600 416 SNTPFMFYNWKVNGVPGEVAVNGILKVGFNIMPTDPYTYGCGVDSADLFRDTGFQLKQEG
(B)BBOV_II006610 417 TESPSMYIQWLLTPLFTTKIREYTFTLMLNILPSDPYTYGCGVDSADLFRKDGFKLSTNT
consensus        421 . .* *. .* .  .           .  **.*.****************  ** *    

(A)BBOV_II006600 476 RGRKVTHCKVNPYLSSPVGFYCPEGFVLEPPNCFSEMLHKDKEVVVPLSDFEPLARALEG
(B)BBOV_II006610 477 EDEDVTECKVNPYLTSPVGFYCPKDHTLEPSNCFEEMINATNNEKVKLSDFAPLARTVES
consensus        481     ** *******.********    *** *** **.       * **** **** .* 

(A)BBOV_II006600 536 RHIKVADFHTSTSNRDHIRYSSVELMCRCLDKEGRVRASITLNLQRPSNLR--RQWNDML
(B)BBOV_II006610 537 KNIRIVDFNLPKKMKKEIIYNKDMLSCRCRDKNGEVRARIVLDLRNPMDSEVIEEYAD--
consensus        541 . *.. **      .  * *    * ***  ** * *** * * * *    ..  . *..

(A)BBOV_II006600 594 KPTRF
(B)BBOV_II006610     -----
consensus        601 .....
```
