# Supplementary material for: Expression of 6-Cys Gene Superfamily Defines Babesia bovis Sexual Stage Development within Rhipicephalus microplus
Source: PLoS One. 2016 Sep 26;11(9):e0163791. doi: 10.1371/journal.pone.0163791 (PMC5036836; doi:10.1371/journal.pone.0163791)
Supplement: S5 Fig — A conserved motif identified in the 6-Cys SF1 proteins is pointed out by red box. Residues depicted in white font over black background indicate conserved amino acids. (PDF) [file pone.0163791.s005.pdf]

```
(E)BBOV_II006640  -MKRNIVHNTTLITAAVA-LALFHNA-TDGVYAAKGINDT
(D)BBOV_II006630  MVSQLHQNGSYLSLFTFS---LLFTL--LKISNVE--NIT
(C)BBOV_II006620  -MKQLRVSNIPLLVYGVTIFTIYSSMPRWLAGAVNPPSLP
(A)BBOV_II006600  -MDIQNTLNRL----ALACFIV-FCKNLLIATATPSIHLD
(B)BBOV_II006610  -MSQLNLLNIF----SITFLSVFLTTSSLCSTATPSIHLD

(E)BBOV_II006640  ITNAFRAVNEYVFEAKTIQYVKPEDFEPEDDILRVVDLYP
(D)BBOV_II006630  LSHVTTS-QKTGKTNRVDFFSNETDLNPGDNLHQVVRLEK
(C)BBOV_II006620  ------------SLRDDKSFVKDE-----------TYLAP
(A)BBOV_II006600  LSH---------NNKQYDDFSVNE-----------VIVAG
(B)BBOV_II006610  LSH---------NNKQYDDFSVNE-----------VIVAG

(E)BBOV_II006640  GDSLKYSCGKEASSTMGTFMLHPSNPKTHILLPKGDEPVD
(D)BBOV_II006630  GDTLFFTCGSDDIFKNGTVERFPKNPLKYTLPSQGEYEGT
(C)BBOV_II006620  GAHLSFVCGSGKD-PEGAYTMYPSNPQERLLLPIEDEDFE
(A)BBOV_II006600  PKEVVITCGNGRD-EDVEHTMYPSDPVSKMLLPPEGNDFT
(B)BBOV_II006610  PKEVVITCGNGRD-EDVEHTMYPSDPVSKMLLPPEGNDFT

(E)BBOV_II006640  VASNRVYPNHTIYRSDRELVISTFRSMGLDHLLIDYARDT
(D)BBOV_II006630  KGLAHELPNHNIFRSDVDIISSYEDEHNGTRLRIKYPSSA
(C)BBOV_II006620  SAVLKEVALRDRFRSDGLTIKFSRGGHEDNYINIHYGING
(A)BBOV_II006600  SAVEKEVASHSFYRSSDLNFEVKKAKDVPVSVKISRTVDT
(B)BBOV_II006610  SAVEKEVASHSFYRSSDLNFEVKKAKDVPVSVKISRTVDT

(E)BBOV_II006640  KIVAKDNKNFSLNLLCIFIPSDVNGSAKFRWLQINFKNVL
(D)BBOV_II006630  VIMAKRPDNFTLNYACKYQPHDDTKPASFKFLEVRFDDVY
(C)BBOV_II006620  VLLAKDPENFSLNLACKYMPRNKNEKPFYRWLKVRFDALY
(A)BBOV_II006600  LIMAKDPENFSLNFACKYQSKDGSNAPVYKWVTIKFEAVY
(B)BBOV_II006610  LIMAKDPENFSLNFACKYQSKDGSNAPVYKWVTIKFEAVY

(E)BBOV_II006640  PIPYGCCSAGYHMFKNSIPIGPDTPHDKVLEAAQCEIEAE
(D)BBOV_II006630  PMAYGCESSDSALFLNGIPQEDESISIKR-QRRLCYIDPI
(C)BBOV_II006620  PMAYGCETGNNMLFKNTAPFVPGFKVSDV-DRVDCSLTLE
(A)BBOV_II006600  PMAYGCETGNNMLFKNSRPIIPNILNL---QIASCSVDIE
(B)BBOV_II006610  PMAYGCETGNNMLFKNTIPKLDWLNHDD--VEEECLITLE

(E)BBOV_II006640  PNMILGIYCSKDDYVYPDDCFRQVIGMD-GKRIMFNQ---
(D)BBOV_II006630  PNMIIGIYCKPGDHLYPSRCFQEAVLDAQGFTIRFNKSYI
(C)BBOV_II006620  PGMIFGIYCKPGEQLMPDHCFPGKRLQELNGDITPYANE-
(A)BBOV_II006600  PGMIFGIYCKAGERLDPGECFSDEELSDYNGAITPYIPKF
(B)BBOV_II006610  PNMLFGIFCEKGERIWPKNCINDDDYKKMNHAISSYSLPW
```

```
(E)BBOV_II006640  FREYSFPHNLIDGNMRLMKLSPKAFNHDMSFSCQCRNKDD
(D)BBOV_II006630  DP--DFPYQEFPERFRLIKLSH-DFDKKAPISCSCVDRNG
(C)BBOV_II006620  -TEFYDSRRKINSRFQLFVVSDKGVRTHFYHHCYCQGPQG
(A)BBOV_II006600  AAN-ANPASTLSTRFRLFKVHDGQLPNSVDLSCACVGAYD
(B)BBOV_II006610  SPTVYRSMSRLSPRFKLFQVSPNEIVKNINIHCYCQNKER

(E)BBOV_II006640  KITSIMKVNLRKTETCDFVQIMDIYRRYGNW--PRKVCRK
(D)BBOV_II006630  KMTKKLIIEKPKNAEVNIMNL--VNRGIVSLNKPPYLLSY
(C)BBOV_II006620  STKVLSIVNHIHSHILPQEYMAQHVTHPQ----ETRYVVY
(A)BBOV_II006600  KTTRILYVNMLGNVVVNASK--QTNNMKADLMPTIKEVIM
(B)BBOV_II006610  ITRKITIKKLVNKSIDFYRLLSTADKNDAS----VFHRME

(E)BBOV_II006640  TLSTNKVIKIIIPKSDGIANHRNDAGGLLLYPENFGVVAY
(D)BBOV_II006630  ILSPGMHLTFLVN-NSSIKLRNGEEAYGWISPKNAVQDIG
(C)BBOV_II006620  NLYPGMMYHVVAPWRDWLDVGLLGKVRQRMVPTNFQK-IY
(A)BBOV_II006600  NMNPGNNITVKYVPDGEVFLSRGRKVTGQLRPTNPDTTAF
(B)BBOV_II006610  YLEPGSKNGFNIPLSGTFKLGSFGMVSGRIFPSNLTYNAY

(E)BBOV_II006640  NPTTNMSHLREIRINRIIGYVGLKMNKIENINNYTFEFST
(D)BBOV_II006630  MIA-DDGKLVEIPLSDAIGSKGFYVGRTELYNGVSYRFVY
(C)BBOV_II006620  TGN-PYRYNDALHFNDIFGSKHFDVLTKVMGPDRIRSLIY
(A)BBOV_II006600  NGL-PVESNPPTPIKDFIGSRGVDITYTKVKDKMVYKVKY
(B)BBOV_II006610  YSL-GSSPFVLSRLRQFIGYDGVGISYTKYKGKMKYTFNY

(E)BBOV_II006640  TENSVIVMKRRIASLSYLYEYYDRFSGPLTKKNTMITIDI
(D)BBOV_II006630  DDNGIVVLKKPDRVISYGWVMLDSRNNARTHLGLALAMYI
(C)BBOV_II006620  RSNGIVVLKVDTPLLYYDWVLKRHHGVR-VDANMRLLFYL
(A)BBOV_II006600  ADDALLVLKSNTPFMFYNWKVNGVPGEVAVNGILKVGFNI
(B)BBOV_II006610  KKDSTLVAKTESPSMYIQWLLTPLFTTKIREYTFTLMLNI

(E)BBOV_II006640  VPTDPYTYGCGAENPDIFNTKGVVFNNQHIQKGAHYHTEV
(D)BBOV_II006630  VPTDPYTYGCGVDSADLFHKEGFLLSFEYDQ-----VPVT
(C)BBOV_II006620  MPTDPYTYGCGVESTDLFRTDGFQISKQEEH-----VSIT
(A)BBOV_II006600  MPTDPYTYGCGVDSADLFRDTGFQLKQEGRG-----RKVT
(B)BBOV_II006610  LPSDPYTYGCGVDSADLFRKDGFKLSTNTED-----EDVT

(E)BBOV_II006640  KCTLNAWKNSPIGFYCPKQYVLEPADCFNSAYLVSTNHVV
(D)BBOV_II006630  KCKVNPYLSSPVGFYCPEGFVLEPPNCFSEMLHKDKEVVV
(C)BBOV_II006620  KCKINPYLTSPVGFYCPKDHTLEPSNCFEEMINATNNEKV
(A)BBOV_II006600  HCKVNPYLSSPVGFYCPEGFVLEPPNCFSEMLHKDKEVVV
(B)BBOV_II006610  ECKVNPYLTSPVGFYCPKDHTLEPSNCFEEMINATNNEKV
```

```
(E)BBOV_II006640   RLDEYAPQGRVLNSPNLRIIDFTGPKSVKYTKKYLEESLQ
(D)BBOV_II006630   PLSDFEPLARALEGRHIKVADFHTSTSNRDHIRYSSVELM
(C)BBOV_II006620   KLSDFAPFARSVESTNLKVADFHMSPRMKKDTKYTDTELK
(A)BBOV_II006600   PLSDFEPLARALEGRHIKVADFHTSTSNRDHIRYSSVELM
(B)BBOV_II006610   KLSDFAPLARTVESKNIRIVDFNLPKKMKKEIIYNKDMLS

(E)BBOV_II006640   CRCRRRDGTVMATITIDLGRPRDK---------------
(D)BBOV_II006630   CRCLDREGKVHASITLDLRKPRTRQL-GNINKAF-----
(C)BBOV_II006620   CSCIDKEGNTRAVITLDLRNPMDPAVIGEYA--------
(A)BBOV_II006600   CRCLDKEGRVRASITLNLQRPSNLR--RQWNDMLKPTRF
(B)BBOV_II006610   CRCRDKNGEVRARIVLDLRNPMDSEVIEEYAD-------
```
