# Supplementary material for: Expression of 6-Cys Gene Superfamily Defines Babesia bovis Sexual Stage Development within Rhipicephalus microplus
Source: PLoS One. 2016 Sep 26;11(9):e0163791. doi: 10.1371/journal.pone.0163791 (PMC5036836; doi:10.1371/journal.pone.0163791)
Supplement: S6 Fig — A partially conserved motif identified in the 6-Cys SF2 proteins is pointed out by red box. Residues depicted in white font over black background indicate conserved amino acids. (PDF) [file pone.0163791.s006.pdf]

```
(I)BBOV_IV007390   -------------------MWIPAIVVLLAIIGNQE-FLI
(J)BBOV_IV007480   --------------------MVSYITVALFIATSVIHLAL
(F)BBOV_II001180   ----------------------------------------
(G)BBOV_II001190   MFVTMSSLRILSSQATKLVLYVISALISLVINTNVC----
(H)BBOV_II001120   --------------------MISFSLVAALLAVNVT----

(I)BBOV_IV007390   KGYHAGKGSVIKWVGNERQANWTINAKWDDKIHYYFDIDI
(J)BBOV_IV007480   AEYD--THEVYDFSNF--------EYPIDNNTVKVYVTVL
(F)BBOV_II001180   --------MLCNFDEF---------ASINDHAAVVCAAKS
(G)BBOV_II001190   ---S--ADVVHDFMDD---------PAITNFAAVICVLNS
(H)BBOV_II001120   ---G--IDILCDFSSE---------APLEENAVLMCVAQS

(I)BBOV_IV007390   YENENVEIRCPNNTSA-EVELVPFKEGM--CYNTDAFGNV
(J)BBOV_IV007480   NSSKSIEILCPKERNGLRFNLLPNTRETIATNDIYVYATV
(F)BBOV_II001180   SREMTVSIKCPKLVGDVEYSWHPHVINPHTCNV-KAYVKV
(G)BBOV_II001190   HNDRFVSISCPDRVGDVNYTWHPRSSNDSYIER-NAYISS
(H)BBOV_II001120   STDRSIYAICPLNVNGVPYGWHPISGNYEN-GD-GVYVSG

(I)BBOV_IV007390   GDGIKEVGV-------------------------------
(J)BBOV_IV007480   NGVYRKININQIFVPDES--------SDSGL---------
(F)BBOV_II001180   GKDLADVNLSDVLKSEDNEPIWRSEEHLNYHILRITTRYN
(G)BBOV_II001190   QHGLETRPVSEIIISESVAPIWIFDRGINSTSFNIYVQRD
(H)BBOV_II001120   TSHLEESSLSRMLVSEYKNKLWHFEVHGSNVILYINVPED

(I)BBOV_IV007390   ----------------------------------------
(J)BBOV_IV007480   PDYRLNKARLLLANDGHTLFRRPGFDSIDVHCTAGIFINS
(F)BBOV_II001180   YHYVMGEDKLMFLCAPKDLDFNSTL---------T-----
(G)BBOV_II001190   EHYIMTENRLMLLCGPVDLELTPTL---------V-----
(H)BBOV_II001120   EHFVMKDNRLLFICAPSTFKLTSSL---------N-----

(I)BBOV_IV007390   ----------------------------------------
(J)BBOV_IV007480   VAASLPGCLDNEQLNRSGAY----TCPCYGCGKASGPLLG
(F)BBOV_II001180   --SYILNDID---IDRSHVIDWMDSATLYNELNERKTGLG
(G)BBOV_II001190   --SYLTNTVD---LDHSHTIRRNTYSSLTRALDKYSSGIG
(H)BBOV_II001120   --YHLTRKID---ITKSIRIPWRDGSGLTEALKPFGKGIG

(I)BBOV_IV007390   ----------------------------------------
(J)BBOV_IV007480   VVKIMLTSVPEVMHGCGSKRVPILLHEQ-------PSSHE
(F)BBOV_II001180   FFFMNRDYFQQPLQGCGSRSSSLFLDKQLVDVDADTGVRS
(G)BBOV_II001190   VVYLRRNSFHHPLMGCGSRASPLFRNPNDVIIDEATGLRT
(H)BBOV_II001120   MLYLRRSNFQQPLQGCGSRSSSLFLDKQLVDVDADTGVRS
```

```
(I)BBOV_IV007390   -------------------ENLDRMKLKISNIAS-NIKFA
(J)BBOV_IV007480   CSYDLLDTDRIGFYCKGRVDPPDCPRVMFNALNDQLMDVP
(F)BBOV_II001180   CEVDPMSSTPIGFLCEGRMEPPECMKYLIDTNGKI----R
(G)BBOV_II001190   CVVDPMSTLPIGFLCEGEIEPPDCFRYLISDNNYV----H
(H)BBOV_II001120   CEVDPMSSTPIGFLCEGRMEPPECMKYLIDTNGKI----R

(I)BBOV_IV007390   ENIKGRWMFMFDGEAYHYGME----TIF----FDCIVNTT
(J)BBOV_IV007480   FEGNLNWQMYGDGLLLSISYDQSCNAKTFKGYCKCISEET
(F)BBOV_II001180   PNRTERWTLMNRSTLVVAQPLTYLATSLFEGHCLCIDPLT
(G)BBOV_II001190   HPLVHRYMTAYNDTLLIVQPFVYLASPLIEGYCVCRDRVN
(H)BBOV_II001120   PNRTERWTLMNRSTLVVAQPLTYLATSLFEGHCLCIDPLT

(I)BBOV_IV007390   VPLHVATIQLNIMPYYKNIPRS--VHMYDFTKN-------
(J)BBOV_IV007480   GAT-N--AVINLRRQDFYVCDLGSLLMRHLSNNILGPWCD
(F)BBOV_II001180   DRV-L--AKIVVKPRYEYVCDINNMLMKNRVQPIHSFWCS
(G)BBOV_II001190   SEV-V--AKLIVKPRYEYVCDINNMLMKNRLKYIRHFWCS
(H)BBOV_II001120   DRV-L--AKIVVKPRYEYVCDINNMLMKNRVQPIHSFWCS

(I)BBOV_IV007390   NYSPPGKFVSYYKFPRPGSAFYVKCSHQGL----------
(J)BBOV_IV007480   VKLYPGRKLSIILPSLNESNVYTNPVTK------------
(F)BBOV_II001180   VTLHPGSTLTIRFPPDPNIILSDTTNTSTL----QAVYPY
(G)BBOV_II001190   FVLHPGSNVTIKFPADSDIILDDDGDEQIELKDDTPPNLF
(H)BBOV_II001120   VTLHPGSTLTIRFPPDPNIILSDTTNTSTL----QAVYPY

(I)BBOV_IV007390   ----------------------SLEA-----------TVG
(J)BBOV_IV007480   --------VAHKVVVKSALWMPLISSNNGLVRSIFNNAVT
(F)BBOV_II001180   ETQFKPSTLEHLHSYTGTTWNKRLE------SVPY-----
(G)BBOV_II001190   YTTFNPSSLRKLRILGSSKWNNHVL------KRHY-----
(H)BBOV_II001120   ETQFKPSTLEHVSFLTGRKWFNSLR------MVDY-----

(I)BBOV_IV007390   LKQYS---IKSGGCEDLSEAADDENVNLCLNFADET----
(J)BBOV_IV007480   YKQVSIEPFFNNGILELESGYDPLGVLIIKHLNNET-RLK
(F)BBOV_II001180   ------HDRIVGDALELDLSMSDRGLIRIIYMSGKPLGTI
(G)BBOV_II001190   ------SEKLAGDALTLDVSNARKGVVKLSYDINKPLTMI
(H)BBOV_II001120   ------NTKLSGDALSMDTSSAHKGVIKIKYHRDKPLAMI

(I)BBOV_IV007390   ---------------------------------IVVLWPY
(J)BBOV_IV007480   MGFD--SFYYRNLMKIGIENGKPMDILANIKITLQQLNGY
(F)BBOV_II001180   KDYDSILFHWHSVPFNKL---IDSMISASISVQLVPTHIY
(G)BBOV_II001190   GSYDSLCFYWRLLPRVNY---PFEHIFATIRITLSLTHPY
(H)BBOV_II001120   GEYEPLCFYWQLLPHRNN---VFGKIITPIKVALAVTHPY
```

```
(I)BBOV_IV007390  N D I K V C S G D K N I F T F V R Q D K D N T K H I H W T Y M L E - - - V F D S
(J)BBOV_IV007480  K W L D E D L H H Y M E D K P I - - - - - A S G R N P W I L R H S F S P F I K A
(F)BBOV_II001180  D T T G C D I G Q P S V F N P R - - - - N M A K Y I R P K E Y I G N T G L M H D
(G)BBOV_II001190  D V V G C E T G E M R I F D P Q - - - - S A R R R C V W K S Y T G I R R F Y R Q
(H)BBOV_II001120  D I K G C D S H Q T P I F D P S - - - - L V A K D C V W K K Y E Y I N G F L H K

(I)BBOV_IV007390  H I D S M D P A K D K I A E F Y H A E D N Y I I A P S K C P E T V Y D R S D I Q
(J)BBOV_IV007480  F - - - - - Y A G D I D V T T V R C N S T E A L V P S G C N K K A F D A N R - -
(F)BBOV_II001180  C V - - V K N F F G V F H T G I Y C G S G N T L M P N G C K Q T A Y S L Y S - -
(G)BBOV_II001190  C I - - I H H N S G V I R T G I Y C K P G E M L M P P N C S E S A F D L S T - -
(H)BBOV_II001120  C V - - L E N A Y G A I K A G I F C K D G E S L M P N N C L E N L Y D Y S L - -

(I)BBOV_IV007390  I D L T D F L T G A I F T R K S T G Y V H I D I S K Y Y T Y T S H F V S C Y K S
(J)BBOV_IV007480  - - - - - - - - - - - - - - - - - - - - - - - - - - - - - - - - - - - - - - - -
(F)BBOV_II001180  - - - - - - - - - - - - - - - - - - - - - - - - - - - - - - - - - - - - - - - -
(G)BBOV_II001190  - - - - - - - - - - - - - - - - - - - - - - - - - - - - - - - - - - - - - - - -
(H)BBOV_II001120  - - - - - - - - - - - - - - - - - - - - - - - - - - - - - - - - - - - - - - - -

(I)BBOV_IV007390  T K R D I K Q P N L T F K F Y P V C D Y E N K D N L D M D G R T C S I F I L N E
(J)BBOV_IV007480  - - - - - - - - - - - - - - - - - - - - - - - - - - - - - - - - - - - - - - - -
(F)BBOV_II001180  - - - - - - - - - - - - - - - - - - - - - - - - - - - - - - - - - - - - - - - -
(G)BBOV_II001190  - - - - - - - - - - - - - - - - - - - - - - - - - - - - - - - - - - - - - - - -
(H)BBOV_II001120  - - - - - - - - - - - - - - - - - - - - - - - - - - - - - - - - - - - - - - - -

(I)BBOV_IV007390  D T V N V R G A A K Y G T P L D L L P S N D S S N Y L K D V T P P H S P W D S T
(J)BBOV_IV007480  - - - - - - - - - - - - - - - - - - - - - - - - - - - - - - - T M V I P F P E G
(F)BBOV_II001180  - - - - - - - - - - - - - - - - - - - - - - - - - - - - - - - N T E E P I P S S
(G)BBOV_II001190  - - - - - - - - - - - - - - - - - - - - - - - - - - - - - - - G G S L P W P P S
(H)BBOV_II001120  - - - - - - - - - - - - - - - - - - - - - - - - - - - - - - - G K T T R L P Q S

(I)BBOV_IV007390  I L N L A H D E G Y G I R F E S I H D K H R D E I H V K F - - - N Y S G F A H T
(J)BBOV_IV007480  V S Q - R M R D P R G N S T Q I V V A D R A S P L S L S C S C L N S D G I E S S
(F)BBOV_II001180  V V R L D L L H V E G M Q M F K I L T N T E T I F S M S C N C V D P R G F V I S
(G)BBOV_II001190  I T N M H M R K V D G L Q M F K F T I N P I E V F S L S C N C I T D R G I A T S
(H)BBOV_II001120  V K S L T Y A K V S G M R I L Q L S Y N N T R P L S V G C S C V D S Q G Y D R S

(I)BBOV_IV007390  P - - - - - - - - K Y F V W Q K G P F D D R N H H E K M M A V H L A D - - A W V
(J)BBOV_IV007480  R F E V H K L R R L D V A - - - N P F L N G A P N R I V M L P H V E M I S N G V
(F)BBOV_II001180  R L I L E N P F S E T I G L F I R P Y N E C L N Y R D Q H L P Y I D - - - G S M
(G)BBOV_II001190  Q L I L E S T Y H E H N V L L I R P L F S A Q R N E R Q - - I Y V N - - - K S D
(H)BBOV_II001120  Q L I L Q S N V Q H M L V L P I I P I P E L N G D M T M K L Y N L G - - - K L T
```

```
(I)BBOV_IV007390  STTESGTLDTESIDKVIADFPNSSKIIQVMRQN-------
(J)BBOV_IV007480  ASMRYGI--------------DRIPMIDSLEAKHEFRLAL
(F)BBOV_II001180  VSYTRGS--------------VPSKTIPLLAQHEKFYLYP
(G)BBOV_II001190  IIYDPSP--------------SDDFLVSIKVNHEGRSISP
(H)BBOV_II001120  ISDARSP--------------GGSHNIDIIGQYEKIVVAR

(I)BBOV_IV007390  TRHVYIDCKSFFD---------------------------
(J)BBOV_IV007480  GDELYLRCQPPVDYKYRQRITVASADKPFFSEAYKTILIH
(F)BBOV_II001180  GTKYIFKCQYCTQ---------------------------
(G)BBOV_II001190  GNTYSYECRYVYG---------------------------
(H)BBOV_II001120  GTSYSLECRYSYQ---------------------------

(I)BBOV_IV007390  --------------EPGYNEYLLYP-QGKNTFFSKLGDGKT
(J)BBOV_IV007480  DESTEKEYTMLNDDPSMSQGVWFPANDKS-FFSVVTKGSD
(F)BBOV_II001180  DMFHRDNYEYTNRSLKQLATAWIPRDASTTYFKTEPFGPS
(G)BBOV_II001190  SALSREWSSIFGQRYIEGSNSWLPRNLRWSYFEKIRRGST
(H)BBOV_II001120  NSSHLQQIIDSEEIPDELKTSWIPKKSESTYLYKYITTSA

(I)BBOV_IV007390  DPSELKSVQLQDEF----AVVGISMYKKPEV--NLGVDLE
(J)BBOV_IV007480  RYF--KNRAI-ESTMAISGGFEIATHEKSTADMISRSIFN
(F)BBOV_II001180  IRF--ISANYEDIIMGEKGGLTFEIKEKYG---AYQEHLV
(G)BBOV_II001190  YHL--RGVNYNEYITGEKNTLVVKIKKNIG---EDNEILE
(H)BBOV_II001120  DRL--IKRNYKDVITGSSKVISVTH-KQSN---DSPALLT

(I)BBOV_IV007390  FSFEADSKIW-----AVHKKPIYFVCAKKGYKH-----G-
(J)BBOV_IV007480  IKYPKSSIVISKLG--ESEITFNFICGKVS---PGLDNAD
(F)BBOV_II001180  VTYSRGTILISKDESQPTRVTFYFICGILPFMKPLIKERN
(G)BBOV_II001190  FNYRSSGILISKDPNNANSLSFHYLCGLRPDVGYGLVDRI
(H)BBOV_II001120  FSYRSSGILISKDPNNANSLSFHYLCGLRPDVGYGLVDRI

(I)BBOV_IV007390  -------------QNSHAYIAFDPLYQ----------LGRLY
(J)BBOV_IV007480  KVEKYSGRFRYTNPIHREDRLMEIWGLIKITIPTTDPYVH
(F)BBOV_II001180  RLLLSLGS----TPPSAKSFPYGTYKLLELSVETTDPYVH
(G)BBOV_II001190  SEV-DRER----LNITDLRYPSGTYKLLEMVVVTTDPYVH
(H)BBOV_II001120  SEV-DRER----LNITDLRYPSGTYKLLEMVVVTTDPYVH

(I)BBOV_IV007390  GCGT---RPELFLNEE----GQRNSNTHCVFKIDDRKTVG
(J)BBOV_IV007480  GCGIPGDHDKLFLPDTDILRDRAGKIIGCSVNMKRAGRAG
(F)BBOV_II001180  GCGLGFKGETLFRDDTITYTEPTSGIKSCVIDLNVNDEGG
(G)BBOV_II001190  GCGVTFTGEEIFKPDTVHITDANDGSSGCQVDLSEHRECG
(H)BBOV_II001120  GCGVTFTGEEIFKPDTVHITDANDGSSGCQVDLSEHRECG
```

```
(I)BBOV_IV007390 F F C P S P I - - - - - - - - P D H F L S G D Q T P W K S T S G A M K N E E N P
(J)BBOV_IV007480 F Y C P L P Y R T D P P D C R P M H M P T S R C V - - G S D S S A L K V S K S P
(F)BBOV_II001180 F Y C P P P Y I M E P P E C F H D V F V G D V V T K L N R I S S D L F A F R S S
(G)BBOV_II001190 F Y C P P P Y V I D P P L C F Q E V L V D G V V T Q L S D I S D S M V Y H K S T
(H)BBOV_II001120 F Y C P P P Y V I D P P L C F Q E V L V D G V V T Q L S D I S D S M V Y H K S T

(I)BBOV_IV007390 Y H I M V K C F D R S T R M Q R F L K P R N P I I D F L E P V S P G E V R S L W
(J)BBOV_IV007480 H L F L F T R T P R M E F - - - - - - - - - - - - - - - - - - - - - - - - - - -
(F)BBOV_II001180 H F S L I R - L D R L A N - - - - - - - - - - - - - - - - - - - - - - - - - - -
(G)BBOV_II001190 H F S L L S - L H H S H K - - - - - - - - - - - - - - - - - - - - - - - - - - -
(H)BBOV_II001120 H F S L L S - L H H S H K - - - - - - - - - - - - - - - - - - - - - - - - - - -

(I)BBOV_IV007390 I F S R G Q F V K H A K P I Y T K V L C N C Y N T E G K R V A S I M L T P D F S
(J)BBOV_IV007480 - - - - - - P F N L L R L R P S T F E C H C S T I T G V R I S T I R L T R I - -
(F)BBOV_II001180 - - - - - - N A K E S R K T Y P A L R C R C V T I T G E L V S S I V I I N Y Q S
(G)BBOV_II001190 - - - - - - Q S N Q E P K M S P P L Q C R C L S I T G E V I S T I N I V N Y Y S
(H)BBOV_II001120 - - - - - - Q S N Q E P K M S P P L Q C R C L S I T G E V I S T I N I V N Y Y S

(I)BBOV_IV007390 V D K L K N S T - - - - - - - - - - - - - - - - - - - - - - - - - - - - - - - -
(J)BBOV_IV007480 - - - - - - - - - - - - - - - - - - - - - - - - - - - - - - - - - - - - - - - -
(F)BBOV_II001180 K G L V S K R - - - - - - W E H F D P A I F F S Q P Y V S K A T N N I A A M Q L
(G)BBOV_II001190 N D S F K R P K G L M R V Y R H I L D E - - - - - - - - - - - - - - - - - - - -
(H)BBOV_II001120 N D S F K R P K G L M R V Y R H I L D E - - - - - - - - - - - - - - - - - - - -

(I)BBOV_IV007390 - - - - - - - - - - - - - - - - - - - - - - - - - - - - - - - - - - - - - - - -
(J)BBOV_IV007480 - - - - - - - - - - - - - - - - - - - - - - - - - - - - - - - - - - - - - - - -
(F)BBOV_II001180 T V G N D S S Q L Y K V L Y G Q Q Y H L Y Y I S L Y Y V K P S R S I C I L D E N L
(G)BBOV_II001190 - - - - - - - - - - - - L F G Q - - - - - - - - - - - - - - - - - - - - - - - -
(H)BBOV_II001120 - - - - - - - - - - - - L F G Q - - - - - - - - - - - - - - - - - - - - - - - -

(I)BBOV_IV007390 - - - - - - - - - - - - - - - - - - - - - - - - - - - - - - - - - - - - - - - -
(J)BBOV_IV007480 - - - - - - - - - - - - - - - - - - - - - - - - - - - - - - - - - - - - - - - -
(F)BBOV_II001180 R Q I R R I E L N D I A S I Q R R I L S A E Q T V K T S T A T Y S D V I E L K L
(G)BBOV_II001190 - - - - - - - - - - - - - - - - - - - - - - - - - - - - - - - - - - - - - - - -
(H)BBOV_II001120 - - - - - - - - - - - - - - - - - - - - - - - - - - - - - - - - - - - - - - - -

(I)BBOV_IV007390 - - - - - - - - - - - - - - - - - - - - - - - - - - - - - - - - - - - - - - - -
(J)BBOV_IV007480 - - - - - - - - - - - - - - - - - - - - - - - - - - - - - - - - - - - - - - - -
(F)BBOV_II001180 S T G G E R W L R F T L P S T E E C E A L C K S I Q I R F G I P V Y N Y G S L N
(G)BBOV_II001190 - - - - - - - - - - - - - - - - - - - - - - - - - - - - - - - - - - - - - - - -
(H)BBOV_II001120 - - - - - - - - - - - - - - - - - - - - - - - - - - - - - - - - - - - - - - - -
```

```
(I)BBOV_IV007390  - - - - - - - - - - - - - - - - - - - - - - - - - - - - - - - - - - - - - - - -
(J)BBOV_IV007480  - - - - - - - - - - - - - - - - - - - - - - - - - - - - - - - - - - - - - - - -
(F)BBOV_II001180  V N F R A A L G D K K A G T S A T G S V T N P S P G N Y H L V K D E Y P V S S L
(G)BBOV_II001190  - - - - - - - - - - - - - - - - - - - - - - - - - - - - - - - - - - - - - - - -
(H)BBOV_II001120  - - - - - - - - - - - - - - - - - - - - - - - - - - - - - - - - - - - - - - - -

(I)BBOV_IV007390  - - - - - - - - - - - - - - - - - - - - - - - - - - - - - - - - - - - - - - - -
(J)BBOV_IV007480  - - - - - - - - - - - - - - - - - - - - - - - - - - - - - - - - - - - - - - - -
(F)BBOV_II001180  G G I L S Q L G L S M G D D D R S L G E L P E E F T N D Y P V I I E G T L E S G
(G)BBOV_II001190  - - - - - - - - - - - - - - - - - - - - - - - - - - - - - - - - - - - - - - - -
(H)BBOV_II001120  - - - - - - - - - - - - - - - - - - - - - - - - - - - - - - - - - - - - - - - -

(I)BBOV_IV007390  - - - - - - - - - - - - - - - - - - - - - - - - - - - - - - - - - - - - - - - -
(J)BBOV_IV007480  - - - - - - - - - - - - - - - - - - - - - - - - - - - - - - - - - - - - - - - -
(F)BBOV_II001180  S Y A N Y R S L K T P S M E S F T V Y R V E W Y I S T R R G E S K D F Y S N P C
(G)BBOV_II001190  - - - - - - - - - - - - - - - - - - - - - - - - - - - - - - - - - - - - - - - -
(H)BBOV_II001120  - - - - - - - - - - - - - - - - - - - - - - - - - - - - - - - - - - - - - - - -

(I)BBOV_IV007390  - - - - - - - - - - - - - - - - - - - - - - - - - - - - - - - - - - - - - - - -
(J)BBOV_IV007480  - - - - - - - - - - - - - - - - - - - - - - - - - - - - - - - - - - - - - - - -
(F)BBOV_II001180  S S S D M L Y L H D Y M I G H Y I K L R V S K A V G T G V N R R Y L Y S V T T R
(G)BBOV_II001190  - - - - - - - - - - - - - - - - - - - - - - - - - - - - - - - - - - - - - - - -
(H)BBOV_II001120  - - - - - - - - - - - - - - - - - - - - - - - - - - - - - - - - - - - - - - - -

(I)BBOV_IV007390  - - - - - - - - - - - - - - - - - - - - - - - - - - - - - - - - - - - - - - - -
(J)BBOV_IV007480  - - - - - - - - - - - - - - - - - - - - - - - - - - - - - - - - - - - - - - - -
(F)BBOV_II001180  G P I R L G N I T A H N V L L N V S K D N E L H T V L A K T D D I H A I A T S L
(G)BBOV_II001190  - - - - - - - - - - - - - - - - - - - - - - - - - - - - - - - - - - - - - - - -
(H)BBOV_II001120  - - - - - - - - - - - - - - - - - - - - - - - - - - - - - - - - - - - - - - - -

(I)BBOV_IV007390  - - - - - - - - - - - - - - - - - - - - - - - - - - - - - - - - - - - - - - - -
(J)BBOV_IV007480  - - - - - - - - - - - - - - - - - - - - - - - - - - - - - - - - - - - - - - - -
(F)BBOV_II001180  N R L P K D G V I T K G P M Q Q S T L D I M Q F S S F M T C K E V P V E E H A T
(G)BBOV_II001190  - - - - - - - - - - - - - - - - - - - - - - - - - - - - - - - - - - - - - - - -
(H)BBOV_II001120  - - - - - - - - - - - - - - - - - - - - - - - - - - - - - - - - - - - - - - - -

(I)BBOV_IV007390  - - - - - - - - - - - - - - - - - - - - - - - - - - - - - - - - - - - - - - - -
(J)BBOV_IV007480  - - - - - - - - - - - - - - - - - - - - - - - - - - - - - - - - - - - - - - - -
(F)BBOV_II001180  S V D E Q N D D I A E T V E T P L P H V R V A S H S D I V D S L S G Y T D K S S
(G)BBOV_II001190  - - - - - - - - - - - - - - - - - - - - - - - - - - - - - - - - - - - - - - - -
(H)BBOV_II001120  - - - - - - - - - - - - - - - - - - - - - - - - - - - - - - - - - - - - - - - -
```

```
(I)BBOV_IV007390   - - - - - - - - - - - - - - - - - - - - - - - - - - - - - - - - - - - - - - - -
(J)BBOV_IV007480   - - - - - - - - - - - - - - - - - - - - - - - - - - - - - - - - - - - - - - - -
(F)BBOV_II001180   I I L N D T D T N E S T I P S S V G I G S S L S S S S E L R S S A T R F N E S L
(G)BBOV_II001190   - - - - - - - - - - - - - - - - - - - - - - - - - - - - - - - - - - - - - - - -
(H)BBOV_II001120   - - - - - - - - - - - - - - - - - - - - - - - - - - - - - - - - - - - - - - - -

(I)BBOV_IV007390   - - - - - - - - - - - - - - - - - - - - - - - - - - - - - - - - - - - - - - - -
(J)BBOV_IV007480   - - - - - - - - - - - - - - - - - - - - - - - - - - - - - - - - - - - - - - - -
(F)BBOV_II001180   L D S I E K E P E G S E D S M A S L L R E P S T K V P V E A P P P K A K S K P P
(G)BBOV_II001190   - - - - - - - - - - - - - - - - - - - - - - - - - - - - - - - - - - - - - - - -
(H)BBOV_II001120   - - - - - - - - - - - - - - - - - - - - - - - - - - - - - - - - - - - - - - - -

(I)BBOV_IV007390   - - - - - - - - - - - - - - - - - - - - - - - - - - - - - - - - - - - - - - - -
(J)BBOV_IV007480   - - - - - - - - - - - - - - - - - - - - - - - - - - - - - - - - - - - - - - - -
(F)BBOV_II001180   L P K V S T P K S A Q G T L K G A I N A K G T A V T G R S P V K P D G T P A V E
(G)BBOV_II001190   - - - - - - - - - - - - - - - - - - - - - - - - - - - - - - - - - - - - - - - -
(H)BBOV_II001120   - - - - - - - - - - - - - - - - - - - - - - - - - - - - - - - - - - - - - - - -

(I)BBOV_IV007390   - - - - - - - - - - - - - - - - - - - - - - - - - - - - - - - - - - - - - - - -
(J)BBOV_IV007480   - - - - - - - - - - - - - - - - - - - - - - - - - - - - - - - - - - - - - - - -
(F)BBOV_II001180   P Q V S K M R G L F K N L I D K G K T L L H R N K G M N A T A I A K N S A N P K
(G)BBOV_II001190   - - - - - - - - - - - - - - - - - - - - - - - - - - - - - - - - - - - - - - - -
(H)BBOV_II001120   - - - - - - - - - - - - - - - - - - - - - - - - - - - - - - - - - - - - - - - -

(I)BBOV_IV007390   - - - - - - - - - - - - - - - - - - - - - - - - - - - - - - - - - - - - - - - -
(J)BBOV_IV007480   - - - - - - - - - - - - - - - - - - - - - - - - - - - - - - - - - - - - - - - -
(F)BBOV_II001180   A K E N I H P K A K H V P N F P K A P A A K K A I P M R D D N L I E V Q L Q L R
(G)BBOV_II001190   - - - - - - - - - - - - - - - - - - - - - - - - - - - - - - - - - - - - - - - -
(H)BBOV_II001120   - - - - - - - - - - - - - - - - - - - - - - - - - - - - - - - - - - - - - - - -

(I)BBOV_IV007390   - - - - - - - - - - - - - - - - - - - - - - - - - - - - - - - - - - - - - - - -
(J)BBOV_IV007480   - - - - - - - - - - - - - - - - - - - - - - - - - - - - - - - - - - - - - - - -
(F)BBOV_II001180   C A G L V I R T T E I E L E L S W P R L D V K E Y C D P S K P P T L P L D P R S
(G)BBOV_II001190   - - - - - - - - - - - - - - - - - - - - - - - - - - - - - - - - - - - - - - - -
(H)BBOV_II001120   - - - - - - - - - - - - - - - - - - - - - - - - - - - - - - - - - - - - - - - -

(I)BBOV_IV007390   - - - - - - - - - - - - - - - - - - - - - - - - - - - - - - - - - - - - - - - -
(J)BBOV_IV007480   - - - - - - - - - - - - - - - - - - - - - - - - - - - - - - - - - - - - - - - -
(F)BBOV_II001180   G I E L R L M Y K R K D S G E S Q L L P L T L R L S S T L Q R S S I D Q G E K D
(G)BBOV_II001190   - - - - - - - - - - - - - - - - - - - - - - - - - - - - - - - - - - - - - - - -
(H)BBOV_II001120   - - - - - - - - - - - - - - - - - - - - - - - - - - - - - - - - - - - - - - - -
```

(I)BBOV_IV007390	- -
(J)BBOV_IV007480	- -
(F)BBOV_II001180	L Q R G L F S D V K E A Y A K I C K Y M G R R N L G
(G)BBOV_II001190	- -
(H)BBOV_II001120	- -
