# Supplementary material for: Expression of 6-Cys Gene Superfamily Defines Babesia bovis Sexual Stage Development within Rhipicephalus microplus
Source: PLoS One. 2016 Sep 26;11(9):e0163791. doi: 10.1371/journal.pone.0163791 (PMC5036836; doi:10.1371/journal.pone.0163791)
Supplement: S1 Table — (DOCX) [file pone.0163791.s007.docx]

**S1 Table:** Primers for gene amplification.

| 6-Cys genes | Forward (5’-3’) | Reverse (5’-3’) |
| --- | --- | --- |
| *A* | **ATGGATATCCAAAACACATTAAATAGG** | **CTAGAATCTCGTAGGCTTTAGCATG** |
| *B* | **ATGTCGCAATTAAACTTAC** | **TTAATCAGCGTACTCCTCTATAACC** |
| *C* | **ATGAAACAACTTCGCGTTTCC** | **CTAGGCGTATTCACCAATAACTGC** |
| *D* | **ATGGTGTCACAACTCCACCAAAATGG** | **TTAAAATGCTTTATTGATGTTCC** |
| *E* | **ATGAAGCGAAATATCGTACACAATACC** | **TTATTTATCACGTGGCCTACCTAGG** |
| *F* | **ATGTTGTGCAATTTCGATGAATTCG** | **TCAGCCTAAATTCGACGTCCCATG** |
| *G* | **ATGTTCGTTACAATGTCATCTCTTCG** | **TTATTGCCCAAACAACTCGTCTAGTATATGACG** |
| *H* | **ATGATTTC TTTTCTCTAGTTGCAGCA CTGCTGGC** | **TTATTGCCCAAACAACTCGTCTAGTATATGACG** |
| *I* | **ATGTGGATACCTGCGATTGTAGTTTTGTTGGCC** | **TCATGTTGAATTTTTCAATTTATCCACCG** |
| *J* | **ATGGTTTCCTACATTACTGTGGC** | **TCAGATTCTTGTTAACCTAATAGTGG** |
