# Supplementary material for: Expression of 6-Cys Gene Superfamily Defines Babesia bovis Sexual Stage Development within Rhipicephalus microplus
Source: PLoS One. 2016 Sep 26;11(9):e0163791. doi: 10.1371/journal.pone.0163791 (PMC5036836; doi:10.1371/journal.pone.0163791)
Supplement: S2 Table — (DOCX) [file pone.0163791.s008.docx]

**S2 Table:** Primes for sequencing.

| 6- Cys gene | Forward 5’-3’ | Reverse 5’-3’ |
| --- | --- | --- |
| ***A*** | **FOR1:ATGGC ATATGGTTGTGAGACAGGA**  **FOR2:ATGCCTACAGATCCCTATACCTATGG** | **R1:ATGTTGAACCCTACTTTCAGTATTCCG**  **R2:CAACATATTATTCCTGTCCACACC** |
| ***B*** | **FOR1:ATGGCATATGGTTGTGAGACAGG**  **FOR2:ATGAAGCGACGGATTGCC** | **R1:GCATATATGTAGATTGACGGAAATCTAC**  **R2:ATGTGGAGTATCCGGGCC** |
| ***C*** | **FOR1:TGACGCTCTATACCCTATGGC**  **FOR2:ATGATATCTTTGGATCAAAGC** | **R1:ATGTAAAGCGTCATTATACCTATAAGG**  **R2:ATGGGTCCTGATAGAATTCG** |
| ***D*** | **FOR1:ATGATACAAAACCAGCTAGTTTC**  **FOR2:ATGCTATTGGAAGTAAAGGG** | **R1:ATGAAATTACTCTGTCAGGC**  **R2:ATGGGGTCTATATAGCAGAGTCG** |
| ***E*** | **FOR1:ATACCTTCAGATGTA AATGG**  **FOR2:ATGAAGCGACGGATTGCC** | **R1:ATGTCAATAGTGATCATCG**  **R2:ATGTGGAGTATCCGGGCC** |
| ***F*** | **FOR1:ATGTCAAGTACACCTATTGG**  **FOR2:ATGGAATAAGAGACTAGAATCTGTGCC**  **FOR3:ATGAGCTGTAACTGTGTTGATCCC**  **FOR4:TATCAAGAACATCTAGTGGTCACATATAGC**  **FOR5:ATGTGTGACAATTACAGG**  **FOR6:ATGAATACCCTGTTTCATCTCTAGG**  **FOR7:ATGACTTGCAAAGAAGTCCCGG**  **FOR8:ATGGAACACCGGCTGTGG** | **R1:TAGCCGTTGCATTCATACCC**  **R2:GTTCATCCACACTGGTAGC**  **R3:CTACGGTCATCGTCGCCC**  **R4:CTACTGTCATTGCCTACGG**  **R5:GTAGACCCTAGTGAGAGC**  **R6:ATGAACAGACCTATCGTTTCG**  **R7:ATGTATATTATACGTATTAATCC**  **R8:ATGCGACCCTCGCAGAGG** |
| ***G*** | **FOR1:ACTTGACTAATACTGTAGATTTGG**  **FOR2:TCTGGTGTTCTTTCGTTCTTCACCCC**  **FOR3: TTGGCCACCATCTATCACC**  **FOR4:TAGATAGGATATCAGAGGTTGACAGGG** | **R1:TCCATATCCCACATCTGGTCGG**  **R2: TTGGTGATAGATGGTGGCCAAGGC**  **R3: ATTAGCATATTATTGATATCGC**  **R4: TAGATATACAACACCAATCCC** |
| ***H*** | **FOR1: TTTAGATAAACAGTTGGTCG**  **FOR2: ATTATCGATGGACACTTCTAGTGCGC**  **FOR3: TTAGTATTGCCTATTATTCCCATTCC**  **FOR4: TACAAACTGTTAGAGATGGTCG** | **R1:TATCCCACATCTGGTCGGAGGCC**  **R2: ATTCTCATACCGCTGACCTTAGCG**  **R3: TACTGCCAGGGTGAAGTGTAACGG**  **R4: TTGAAAATTGCTTCGTCGCAGG** |
| ***I*** | **FOR1:TGGTTGCGAGGATCTATCAGAAGCTGC**  **FOR2: AATGAAGACACGGTTAATGTGAGAGG**  **FOR3: AGTGTTCAACTCCAAGATGAATTTGCC** | **R1: AACGACGGCAAATTCATCTTGG**  **R2: TAGTATATGTATAGTATTTGC**  **R3: AACACTTCACATAAAACGCACTGCC** |
| ***J*** | **FOR1: TCATGAATGTTCATATGATTTGTTGG**  **FOR2: AATAACATTACAGCAATTGAACGGC**  **FOR3: AAGAGTATACCATGTTAAATGATGACCC** | **R1: ATGTCAGCCGTAGACTTCTCG**  **R2: AATGGAATAACCATAGTTCGG**  **R3: TTAGCGTTGCAACTTTGGTCATAGC** |
