# Supplementary material for: Expression of 6-Cys Gene Superfamily Defines Babesia bovis Sexual Stage Development within Rhipicephalus microplus
Source: PLoS One. 2016 Sep 26;11(9):e0163791. doi: 10.1371/journal.pone.0163791 (PMC5036836; doi:10.1371/journal.pone.0163791)
Supplement: S3 Table — Bbo 6-Cys genes from A to J in the Tx attenuated strain, T-virulent and attenuated strains, L17, and Mo7 clonal line. (DOCX) [file pone.0163791.s009.docx]

**S3 Table:** Genbank accession numbers for gene members of the Bbo 6-Cys family in five different strains. Bbo 6-Cys genes from *A* to *J* in the Tx attenuated strain, T-virulent and -attenuated strains, L17, and Mo7 clonal line**.**

|  | Tx attenuated | T-virulent | T-attenuated | L17 virulent | Mo7 |
| --- | --- | --- | --- | --- | --- |
| ***A*** | **KT263532** | **KT263542** | **KT263562** | **KT263552** | **KT263572** |
| ***B*** | **KT263533** | **KT263543** | **KT263563** | **KT263553** | **KT263573** |
| ***C*** | **KT263534** | **KT263544** | **KT263564** | **KT263554** | **KT263574** |
| ***D*** | **KT263535** | **KT263545** | **KT263565** | **KT263555** | **KT263575** |
| ***E*** | **KT263536** | **KT263546** | **KT263566** | **KT263556** | **KT263576** |
| ***F*** | **KT263537** | **KT263547** | **KT263567** | **KT263557** | **KT263577** |
| ***G*** | **KT263538** | **KT263548** | **KT263568** | **KT263558** | **KT263578** |
| ***H*** | **KT263539** | **KT263549** | **KT263569** | **KT263559** | **KT263579** |
| ***I*** | **KT263540** | **KT263550** | **KT263570** | **KT263560** | **KT263580** |
| ***J*** | **KT263541** | **KT263551** | **KT263571** | **KT263561** | **KT263581** |
