# Supplementary material for: Expression of 6-Cys Gene Superfamily Defines Babesia bovis Sexual Stage Development within Rhipicephalus microplus
Source: PLoS One. 2016 Sep 26;11(9):e0163791. doi: 10.1371/journal.pone.0163791 (PMC5036836; doi:10.1371/journal.pone.0163791)
Supplement: S4 Table — Amino acid sequences of the synthetic peptides used for the production of rabbit polyclonal antibodies against the Bbo6-Cys proteins A and B. More than one synthetic peptide was used as a cocktail for the production of antibodies against the proteins. The optical density [OD] values obtained in ELISA reaction testings’ for each of the peptide cocktails using rabbit sera 8 weeks after the start of the immunizations (1:200 dilution) is shown in the table. (DOCX) [file pone.0163791.s010.docx]

**S4 Table:** Amino acid sequences of the synthetic peptides used for the production of rabbit polyclonal antibodies against the Bbo6-Cys proteins A and B. More than one synthetic peptide was used as a cocktail for the production of antibodies against the proteins. The optical density [OD] values obtained in ELISA reaction testings’ for each of the peptide cocktails using rabbit sera 8 weeks after the start of the immunizations (1:200 dilution) is shown in the table.

| 6-Cys protein | Peptide cocktail | Peptide name | Predicted peptide size | ELISA |
| --- | --- | --- | --- | --- |
|  |  |  |  | **OD** |
| **A** | **FQLKQEGRGRKVTHC**  **KVHDGQLPNSVDLSC**  **SNLRRQWNDMLKPTRF** | **AA469-483**  **AA268-282**  **AA583-598** | **17.87 Da**  **16.11 Da**  **21.65 Da** | **1.7**  **0.25**  **2.3** |
| **B** | **KDGFKLSTNTEDEDVTEC**  **CQNKERITRKITIKKLVNKS** | **AA467-484**  **AA287-306** | **20.31 Da**  **24.00 Da** | **1.18**  **>3.50** |
