# Supplementary material for: Expression of 6-Cys Gene Superfamily Defines Babesia bovis Sexual Stage Development within Rhipicephalus microplus
Source: PLoS One. 2016 Sep 26;11(9):e0163791. doi: 10.1371/journal.pone.0163791 (PMC5036836; doi:10.1371/journal.pone.0163791)
Supplement: S5 Table — (DOCX) [file pone.0163791.s011.docx]

**S5 Table:** Genes containing s48/45 domains in: *B. bigemina* [Bond strain], *B. microti* [R1], *Theileria equi* [WA strain], *T. parva* [Muguga strain] and *T. annulata* [Ankara strain].

| Parasite name | Chromosome # | Gene name |
| --- | --- | --- |
| *Babesia bigemina* | I | BBBOND_0110910 |
|  |  | BBBOND_0110570 |
|  |  | BBBOND_0110230 |
|  |  | BBBOND_0109960 |
|  |  | BBBOND_0108200 |
|  |  | BBBOND_0108060 |
|  |  | BBBOND_0108010 |
|  |  | BBBOND_0107660 |
|  |  | BBBOND_0107510 |
|  |  | BBBOND_0107310 |
|  |  | BBBOND_0106590 |
|  |  | BBBOND_0104710 |
|  |  | BBBOND_0104440 |
|  |  | BBBOND_0104060 |
|  |  | BBBOND_0103570 |
|  |  | BBBOND_0103540 |
|  |  | BBBOND_0103530 |
|  |  | BBBOND_0102440 |
|  |  | BBBOND_0102210 |
|  |  | BBBOND_0101930 |
|  |  | BBBOND_0101590 |
|  |  | BBBOND_0101360 |
|  |  | BBBOND_0100690 |
|  |  | BBBOND_0100160 |
|  | II | BBBOND_0201110 |
|  |  | BBBOND_0211950 |
|  |  | BBBOND_0211930 |
|  |  | BBBOND_0210440 |
|  |  | BBBOND_0208600 |
|  |  | BBBOND_0208120 |
|  |  | BBBOND_0207880 |
|  |  | BBBOND_0207460 |
|  |  | BBBOND_0206750 |
|  |  | BBBOND_0205870 |
|  |  | BBBOND_0205830 |
|  |  | BBBOND_0204580 |
|  |  | BBBOND_0204190 |
|  |  | BBBOND_0203340 |
|  |  | BBBOND_0203000 |
|  |  | BBBOND_0202300 |
|  |  | BBBOND_0201670 |
|  |  | BBBOND_0201590 |
|  |  | BBBOND_0201340 |
|  |  | BBBOND_0201020 |
|  | III | BBBOND_0313340 |
|  |  | BBBOND_0312920 |
|  |  | BBBOND_0312910 |
|  |  | BBBOND_0312500 |
|  |  | BBBOND_0311760 |
|  |  | BBBOND_0311490 |
|  |  | BBBOND_0311480 |
|  |  | BBBOND_0310850 |
|  |  | BBBOND_0310520 |
|  |  | BBBOND_0310290 |
|  |  | BBBOND_0307700 |
|  |  | BBBOND_0307350 |
|  |  | BBBOND_0307190 |
|  |  | BBBOND_0306930 |
|  |  | BBBOND_0306900 |
|  |  | BBBOND_0306690 |
|  |  | BBBOND_0306190 |
|  |  | BBBOND_0305950 |
|  |  | BBBOND_0305770 |
|  |  | BBBOND_0303270 |
|  |  | BBBOND_0302630 |
|  |  | BBBOND_0302080 |
|  |  | BBBOND_0300830 |
|  |  | BBBOND_0300450 |
|  | IV | BBBOND_0400600 |
|  |  | BBBOND_0400630 |
|  |  | BBBOND_0400690 |
|  |  | BBBOND_0400770 |
|  |  | BBBOND_0401600 |
|  |  | BBBOND_0402540 |
|  |  | BBBOND_0402900 |
|  |  | BBBOND_0403670 |
|  |  | BBBOND_0404390 |
|  |  | BBBOND_0404940 |
|  |  | BBBOND_0405030 |
|  |  | BBBOND_0405260 |
|  |  | BBBOND_0405710 |
|  | Undefined | BBBOND_0001420 |
| *Theileria annulata* | II | TA14250 |
|  | III | TA03640 |
| *Theileria parva* | II | TP02_0629 |
|  | III | TP03_0268 |
| *Babesia microti* | III | BBM_III00485 |
|  |  | BBM_III08080 |
| *Babesia [Theileria] equi* | I | BEWA_024360 |
